# Introducing or removing heparan sulfate binding sites does not alter brain uptake of the blood–brain barrier shuttle scFv8D3

**DOI:** 10.1038/s41598-022-25965-x

**Published:** 2022-12-12

**Authors:** Andrés de la Rosa, Nicole G. Metzendorf, Jamie I. Morrison, Rebecca Faresjö, Fadi Rofo, Alex Petrovic, Paul O’Callaghan, Stina Syvänen, Greta Hultqvist

**Affiliations:** 1 Protein Drug Design Group, Department of Pharmacy, Uppsala University, Uppsala, Sweden; 2 Department of Public Health and Caring Sciences, Uppsala University, Uppsala, Sweden; 3 Department of Medical Cell Biology, Uppsala University, Uppsala, Sweden

**Keywords:** Proteins, Neurological disorders

## Abstract

The blood–brain barrier (BBB) greatly limits the delivery of protein-based drugs into the brain and is a major obstacle for the treatment of brain disorders. Targeting the transferrin receptor (TfR) is a strategy for transporting protein-based drugs into the brain, which can be utilized by using TfR-binding BBB transporters, such as the TfR-binding antibody 8D3. In this current study, we investigated if binding to heparan sulfate (HS) contributes to the brain uptake of a single chain fragment variable of 8D3 (scFv8D3). We designed and produced a scFv8D3 mutant, engineered with additional HS binding sites, HS(+)scFv8D3, to assess whether increased HS binding would improve brain uptake. Additionally, a mutant with a reduced number of HS binding sites, HS(−)scFv8D3, was also engineered to see if reducing the HS binding sites could also affect brain uptake. Heparin column chromatography showed that only the HS(+)scFv8D3 mutant bound HS in the experimental conditions. Ex vivo results showed that the brain uptake was unaffected by the introduction or removal of HS binding sites, which indicates that scFv8D3 is not dependent on the HS binding sites for brain uptake. Conversely, introducing HS binding sites to scFv8D3 decreased its renal excretion while removing them had the opposite effect.

## Introduction

Immunotherapy is a rapidly growing field where several protein-based drugs have successfully been developed for treatment of a wide range of diseases, including autoimmune diseases, cardiovascular diseases and various cancers^1^. Unfortunately, these advances of immunotherapy have been modest relating to the treatment of brain disorders, likely because of the blood–brain barrier (BBB), which greatly limits delivery of protein-based drugs into the brain^2^.

The BBB consists of tightly connected endothelial cells supported by pericytes and astrocytes. It is a crucial barrier which protects the brain by strictly regulating what substances that are able to reach the brain from the blood circulation^3^. A promising strategy to deliver protein-based drugs across the BBB is receptor mediated transcytosis (RMT), which is the primary mechanism for brain uptake of endogenous proteins such as transferrin^2,4^. When transferrin binds to the transferrin receptor (TfR) endocytosis is induced, followed by intracellular sorting of the endocytosed vesicle^1^. One of the possible outcomes of the sorting is exocytosis at the basolateral plasma membrane, hence transcytosis of transferrin into the brain milieu ^1^. Alternatively, TfR can be recycled to the apical membrane or sorted to lysosomes^1^. The BBB transcytosis mechanism can be utilized by TfR-binding BBB transporters^5^, such as the TfR-binding antibody 8D3^6,7^. The TfR binding capacity of 8D3 has been harnessed to create fusion- and bispecific protein based drugs, with the aim of delivering therapeutics to the brain^8–12^. In additon, a single chain fragment variable (scFv) of 8D3 (scFv8D3) has also been utilized for BBB transport and is the maivn antibody variant used in this study^13–18^.

Several antibodies that bind the TfR have been generated, but most of them are not able to cross the BBB efficiently^5^. In order for a TfR binder to cross the BBB, the bound TfR receptor must be endocytosed, avoid recycling back to the apical membrane and escape lysosomal degradation. To this end, two important properties of TfR binders correlated to BBB uptake have been shown to be affinity to TfR and TfR binding valency^5^. Too high affinity to TfR may result in lysosomal degradation in vivo^19^. Binding of TfR with low affinity at lysosomal pH (pH 5.5) allows TfR binders to escape lysosomal degradation in a BBB cell model^20^. The importance of TfR affinity for brain uptake has further been demonstrated in vivo by comparing monovalently binding chimeric antibodies containing a single TfR binding arm, each with varying TfR binding affinities. The study found that higher affinity constructs had higher brain uptake at therapeutic doses (4 mg/kg), but lower brain uptake at a very high therapeutic dose (20 mg/kg) when compared to lower affinity constructs^21^. The importance of binding valency for brain uptake has been shown by comparing a construct utilizing a bivalently binding double chain antibody fragment (dFab) of 8D3 to a monovalently binding single chain antibody fragment (sFab) variant of the same construct in vivo. The dFab construct was shown to accumulate in brain endothelial capillary cells (BECs) and co-localize with lysosomal markers, while the sFab construct achieved high brain uptake^8^. Unpublished findings from our lab have recently shown that scFv8D3, which binds monovalently to TfR, is more efficient at crossing the BBB at higher concentrations compared to a partly bivalent construct with the same TfR binder. The partly bivalent binder, which has a higher affinity due to the avidity effect, is more efficient at crossing the BBB at lower concentrations.

HS chains are constituents of heparan sulfate proteoglycans (HSPGs), which are glycoproteins with a subset located on the cell membrane surface of most animal cells^22^. HSPGs are abundantly present on all endothelial cells, including brain endothelial cells (BEC) at the BBB^23,24^. HSPGs have a wide range of functions, including regulation of receptor trafficking, regulation of endocytosis and as a binding site for a wide range of ligands^25–27^. HSPGs wide range of functions are enabled by their HS chains^28^, which consist of long, unbranched negatively charged disaccharide repeats, varying in chain lengths and sulphation patterns^27,29^. Binding of proteins to the negatively charged HS require positively charged HS binding motifs^30,31^. Proposed HS binding motifs consist of positively charged amino acids spaced approximately 5–10 Å or 20 Å apart, often consisting of hydropathic, neutrally charged amino acids, in patterns such as XBBXBX, BBBXXBB, or BBXXXBB, where B denotes the positively charged basic amino acids arginine, lysine or histidine^30,32–35^. However, not all HS binding sites are linearly contiguous, as sequence-distant positively charged amino acids may be brought together spatially due to protein folding^32^. One example of this is observed in the protein antithrombin III, which contains a higher order HS binding motif (HS binding cluster) comprised of a linearly contiguous domain and a sequence-remote, positively charged amino acid^33^.

**Figure 1:**
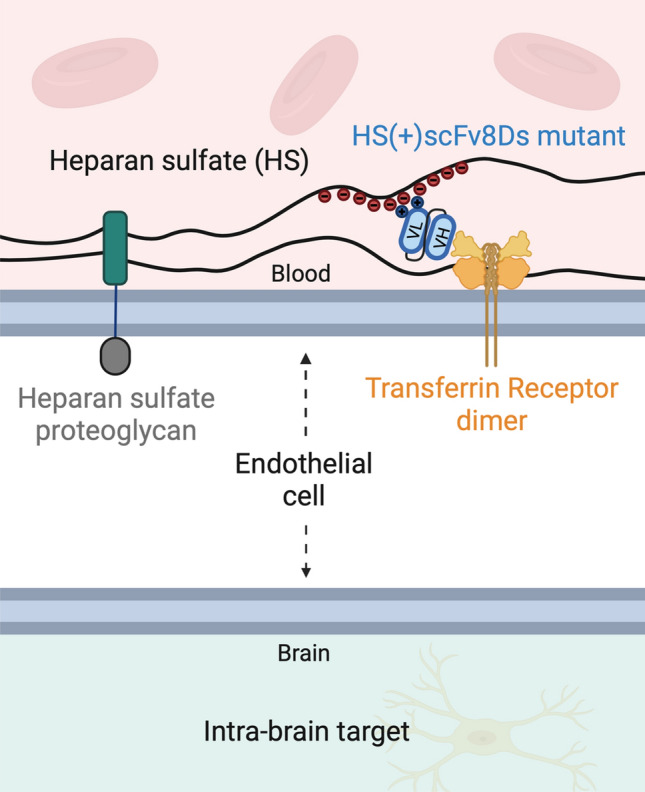
Illustration depicting the hypothetical scenario of HS-mediated brain uptake of TfR binders. This study attempted to increase the brain uptake of the TfR-binder scFv8D3 by introducing extra HS binding sites, creating the HS(+)scFv8D3 mutant (blue).

It has been shown that transferrin binds with ionic interactions to HS^36,37^ and this interaction has been hypothesized to be important for its transcytosis across the liver endothelium^37,38^. This hypothesis is based on the finding that lactoferrin, which has no affinity to TfR, but has high affinity to HS, can inhibit iron uptake by the liver^37,38^. It has been shown that lactoferrin can displace transferrin from HS, which could explain its ability to inhibit iron uptake^37,38^. Further supporting evidence shows that cell surface HS assists receptor mediated endocytosis of HS-bound proteins by presenting the ligand to its receptor and/or stabilizing receptor:ligand interactions^25,26,39,40^. With this in mind, we wanted to investigate if HS binding is important for the brain uptake of scFv8D3. We engineered two scFv8D3 mutants, one with reduced HS binding sites, HS(−)scFv8D3, and one with additional HS binding sites, HS(+)scFv8D3, with the aim to assess the correlation between HS binding sites and brain uptake of scFv8D3 (Fig. 1). Based on the results of the study, we conclude that the modified HS binding sites in both HS(−)scFv8D3 and HS(+)scFv8D3 mutants have a minimal impact on brain uptake.

## Results

### Generation and in vitro characterization of scFv8D3 constructs

**Figure 2:**
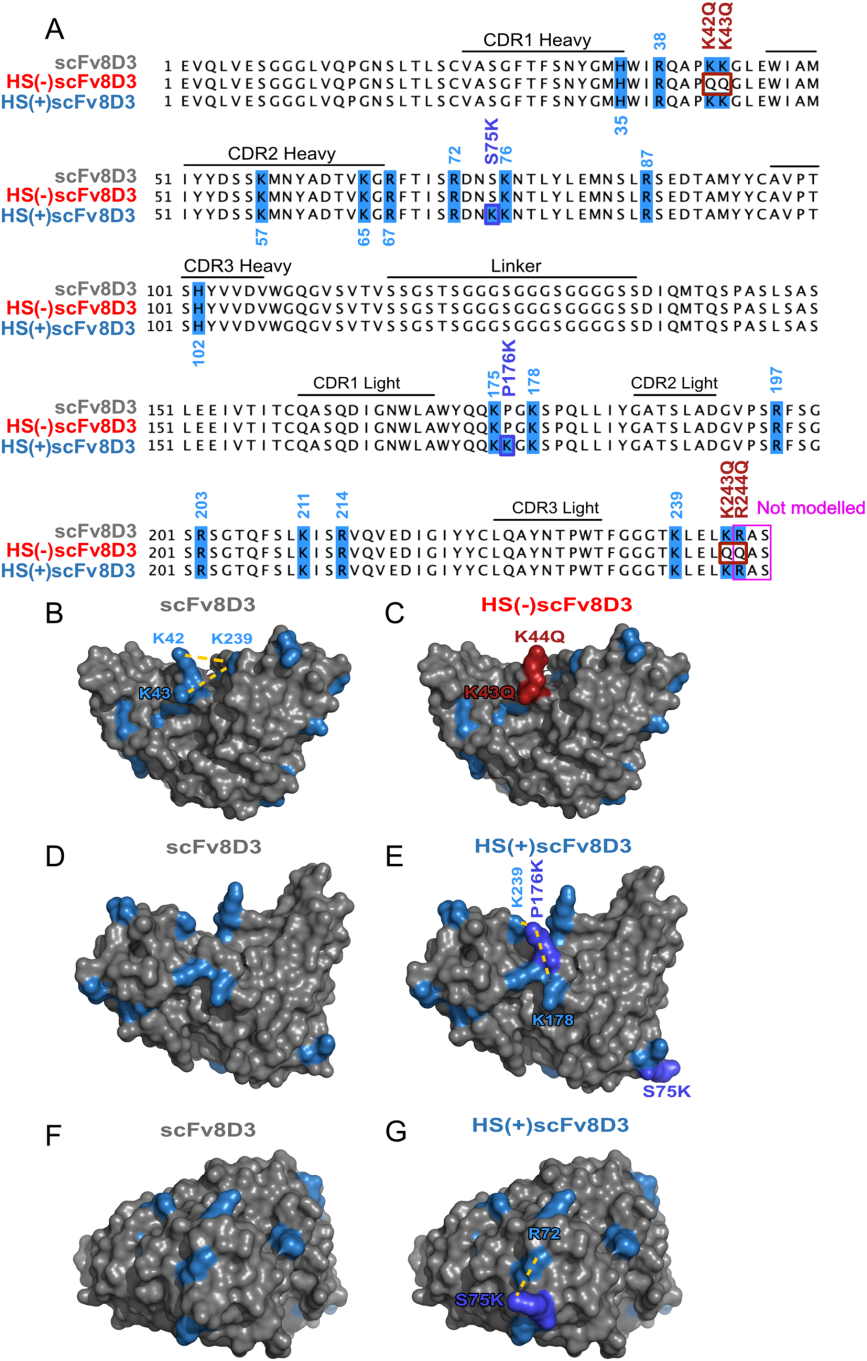
scFv8D3 and HS mutants sequence alignment and structures. (**A**) The amino acid sequences of scFv8D3, the HS(−)scFv8D3 mutant (K42Q_K43Q and K243Q_R244Q) and the HS(+)scFv8D3 mutant (S75K and P176K). Positively charged amino acids are highlighted in light blue. HS(+)scFv8D3 and HS(−)scFv8D3 mutations are highlighted in dark blue and dark red respectively. (**B**) PyMOL model of scFv8D3 with dashed yellow lines show HS binding clusters with 5–10 Å or ~ 20 Å distance between the positively charged amino acids, showing that K42 and K43 is within interaction range to K239. Additionally, K239 is within interaction range of K243 and R244, though this is not depicted due to the exclusion of R244 (**C**) HS(−)scFv8D3 mutant (K42Q, K43Q and R244Q) point-mutations shown in dark red. (**D**) scFv8D3 at the angle where a HS binding cluster was introduced with point mutation (P176K) in the HS(+)scFv8D3. (**E**) HS(+)scFv8D3 (S75K and P176K) mutant showing the new HS clusters added by the P176K mutation (dark blue), where P176K is within interaction range with K239 and K178. (**F**) scFv8D3 at the angle where a HS binding cluster was introduced with point mutation (S75K) in the HS(+)scFv8D3. (**G**) HS(+)scFv8D3 (S75K and P176K) mutant showing a HS binding cluster introduced by the S75K mutation (dark blue) where S75K is within interaction range of R72.

To evaluate if HS-binding affects brain uptake of scFv8D3, variants of scFv8D3 that bind HS with different strengths were created. The HS binding motifs described in the literature^30,31^ are present in linear peptide regions or in nonlinear higher order HS binding clusters^33^, with distances of ~ 5–10 Å or ~ 20 Å between the positively charged amino acid residues ^30,33–35^. The distances of these HS binding motifs and HS binding clusters were used as a template when creating the HS binding clusters on the surface of scFv8D3, based on a homology modeled 3D structure. To avoid disrupting the integrity of scFv8D3, the strategy for creating HS binding sites was to search for positively charged amino acids on the surface, with their R-groups facing outward, where a HS binding motif or HS binding cluster could be introduced with a single point mutation. There were no linear regions on the surface of scFv8D3 where a HS binding motif could be introduced by this strategy. However, two adjacent positively charged amino acids were found in the folded regions of scFv8D3, allowing for the creation of HS binding clusters via the introduction of a third positively charged amino acid at the desired distance. To reduce the HS binding of scFv8D3, point mutations were introduced targeting one identified HS binding cluster (K42Q_K43Q) and one HS binding motif (K243Q_R244Q), generating the HS(−)scFv8D3 mutant (Fig. 2C). Due to the final three amino acids of scFv8D3 at its C-terminal domain (CTD) being excluded by the modelling software when generating the 3D structure, R244, which is part of the HS binding motif K239-XXX-K243-R244, cannot be visualized. However, as the final three amino acids were excluded by the modelling software (Fig. 2A), we make the assumption that the binding motif does not form any secondary structure and that R244 is located on the surface of scFv8D3. To increase the HS binding of scFv8D3, two new HS binding clusters were introduced by point mutations on the surface of the scFv (S75K and P176K) (Fig. 2E,G) generating the HS(+)scFv8D3 mutant. For comparison, similar orientations of the non-mutated scFv8D3 are presented in Fig. 2B,D,F. Finally, to prevent introducing instability in the scFv8D3 mutants, highly hydrophobic amino acids were not mutated into charged amino acids, and vice versa, since hydrophobicity is one of the main forces affecting protein folding^41,42^.

### Production of constructs

**Figure 3:**
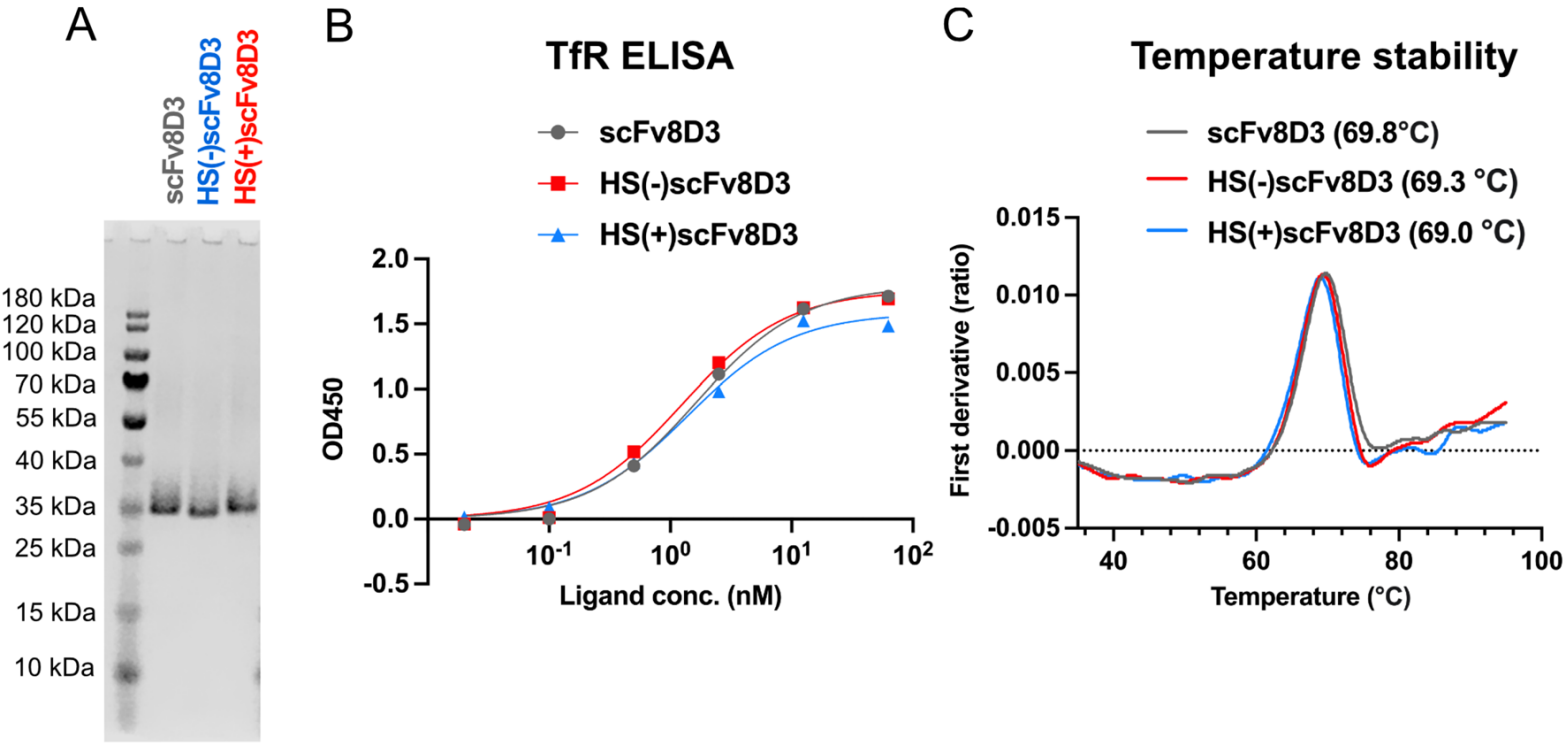
Characterization of purity, functionality and thermostability of scFv constructs. (**A**). SDS-PAGE of the purified scFvs showing a single band at the expected size of 31 kDa, for scFv8D3, HS(−)scFv8D3 and HS(+)scFv8D3. (**B**). Indirect ELISA showing similar scFv binding affinities to TfR. (**C**). Structural stability of the scFvs under thermal stress measured with Tycho nt.6 system starting at 30 °C. Full sized image of SDS-PAGE gel in supplementary Fig. S1.

The three scFv constructs were expressed in Expi293 cells with yields in the range of 2–4 mg per liter of transfected cell culture. SDS-PAGE analysis of the scFvs showed a single band at the expected size of 31 kDa (Fig. 3A), full sized image of SDS-PAGE gel is shown in supplementary Fig. S1. The purity of the scFvs was estimated to be approximately 87% for scFv8D3, 90% for HS(−)scFv8D3, and 92% for HS(+)scFv8D3 (Fig. S1). Indirect TfR ELISA showed that the HS(+)scFv8D3, HS(−)scFv8D3 and scFv8D3 had similar binding affinities to TfR. (Fig. 3B). The scFvs were subjected to thermal stress to test their structural stability. They all exhibited one inflection temperature at 69.0–69.8 °C (Fig. 3C), which correlates to previously reported inflection temperatures for scFvs^43^.

### Evaluation of HS binding capacity

**Figure 4:**
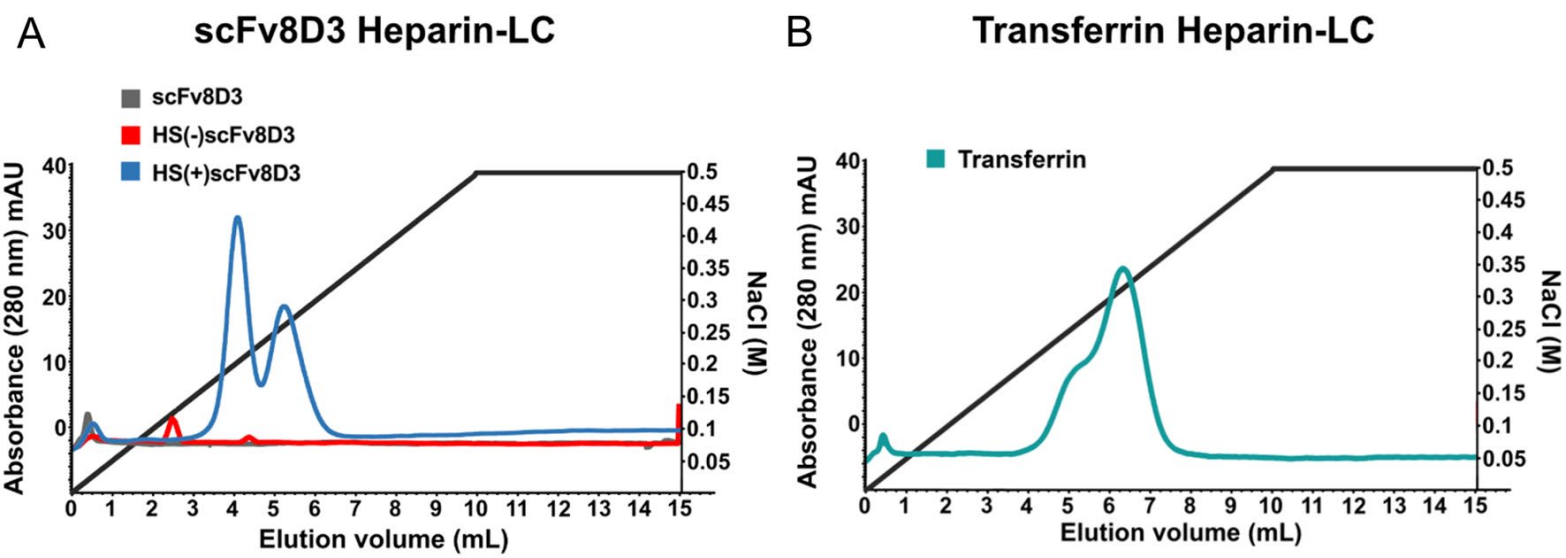
HS binding capacity determined by heparin column chromatography. Heparin binding strength was analysed by heparin affinity LC with gradient NaCl elution (black line). The absorbance was monitored at 280 (nM). (**A**) The HS(+)scFv8D3 mutant (blue) binds to heparin and has one peak at 0.20 M NaCl and second peak at approximately 0.25 M NaCl. The scFv8D3 is shown in grey and the HS(−)scFv8D3 mutant is shown in red. (**B**) Transferrin (teal line) binds to heparin and has an elution peak at approximately 0.25–0.30 M NaCl.

To evaluate the HS binding capacity of the three scFvs, heparin column liquid chromatography (LC) experiments were performed at pH 7. The LCs showed that only the HS(+)scFv8D3 mutant was able to bind to heparin, with two peaks eluted at approximately 0.20 and 0.25 M NaCl. The scFv8D3 and HS(−)scFv8D3 mutant passed through the column without binding, resulting in no elution peaks (Fig. 4A). Transferrin bound to the heparin column, corroborating results from previous studies^36,37^, resulting in one elution peak at approximately 0.25–0.30 M NaCl (Fig. 4B).

### Stability of constructs in vivo

**Figure 5:**
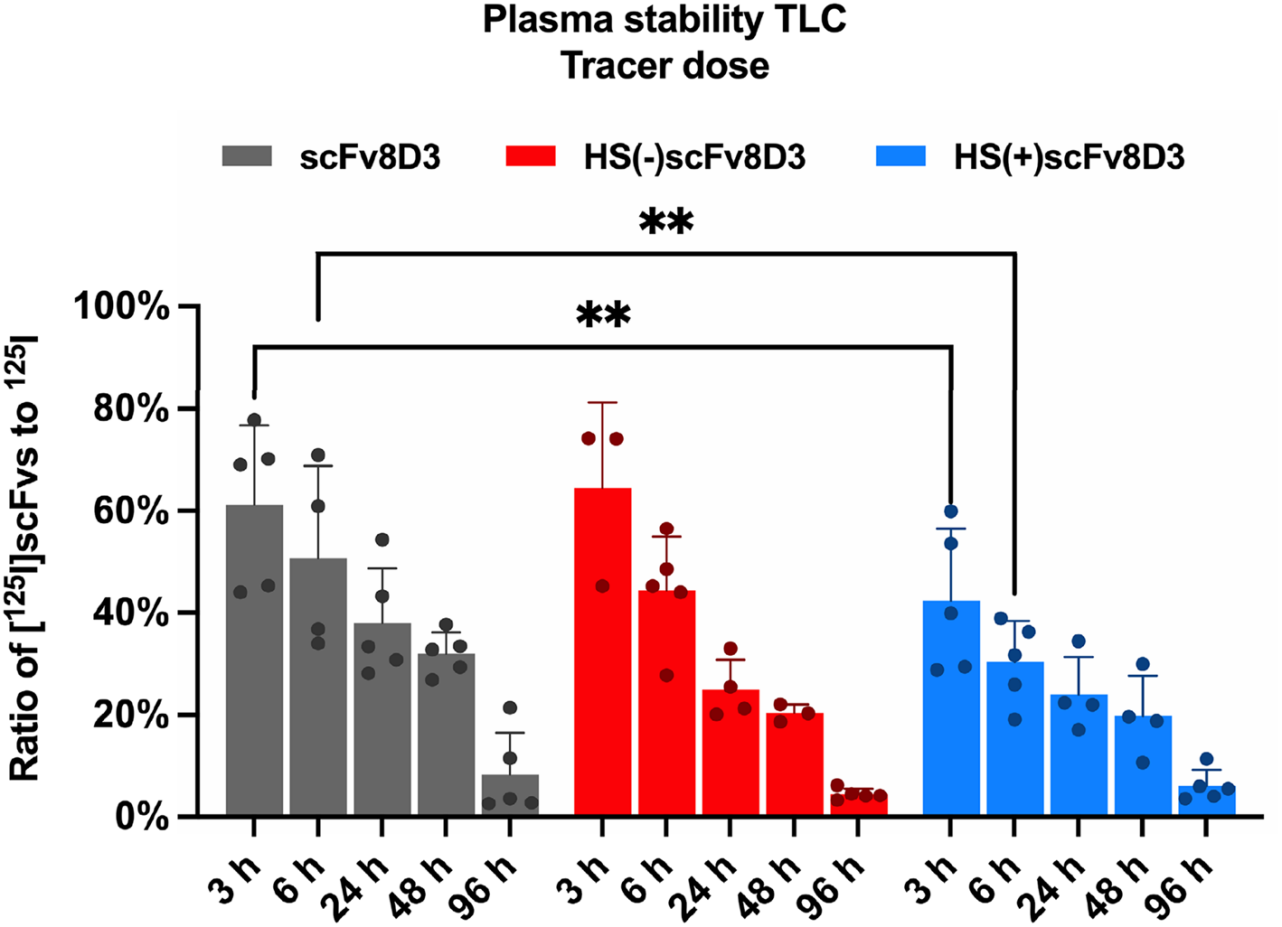
In vivo plasma stability of [^125^I]scFvs constructs. TLC analysis was performed on plasma samples from mice at the indicated times, following an intravenous injection of the ^125^I radiolabeled scFv constructs (0.3 nmol/kg). Semi-quantitative estimation of the ratio between the [^125^I]scFvs to free ^125^I was performed by measuring optical density with Fiji (ImageJ) software^44^. Results are presented as mean ± SD and statistical comparisons were conducted between HS(−)scFv8D3 and HS(+)scFv8D3 to scFv8D3. * Represents a significance of P < 0.05, ** Represents a significance level of P < 0.01.

To assess the stability of the scFv constructs in vivo, they were labeled with iodine-125 (^125^I) and intravenously injected at a tracer dose of 0.3 nmol/kg. Blood samples were taken from the animals and the plasma from each animal was analyzed by thin-layer chromatography (TLC). Three hours post injection, approximately 60% of the ^125^I in the plasma originated from radiolabeled scFv8D3 and HS(−)scFv8D3 constructs respectively, while 40% originated from unbound, free ^125^I. In contrast, for the HS+scFv8D3 construct 60% of the ^125^I was unbound. The fraction of free iodine was significantly higher for HS(+)scFv8D3 compared to scFv8D3 at the three- and sixhour time points (Fig. 5). This pattern persisted together with steady reduction of the radiolabeled scFv constructs at 6-, 24-, 48- and 96 h post injection (Fig. 5).

### Brain uptake of tracer dose of scFv8D3 and HS mutants

**Figure 6:**
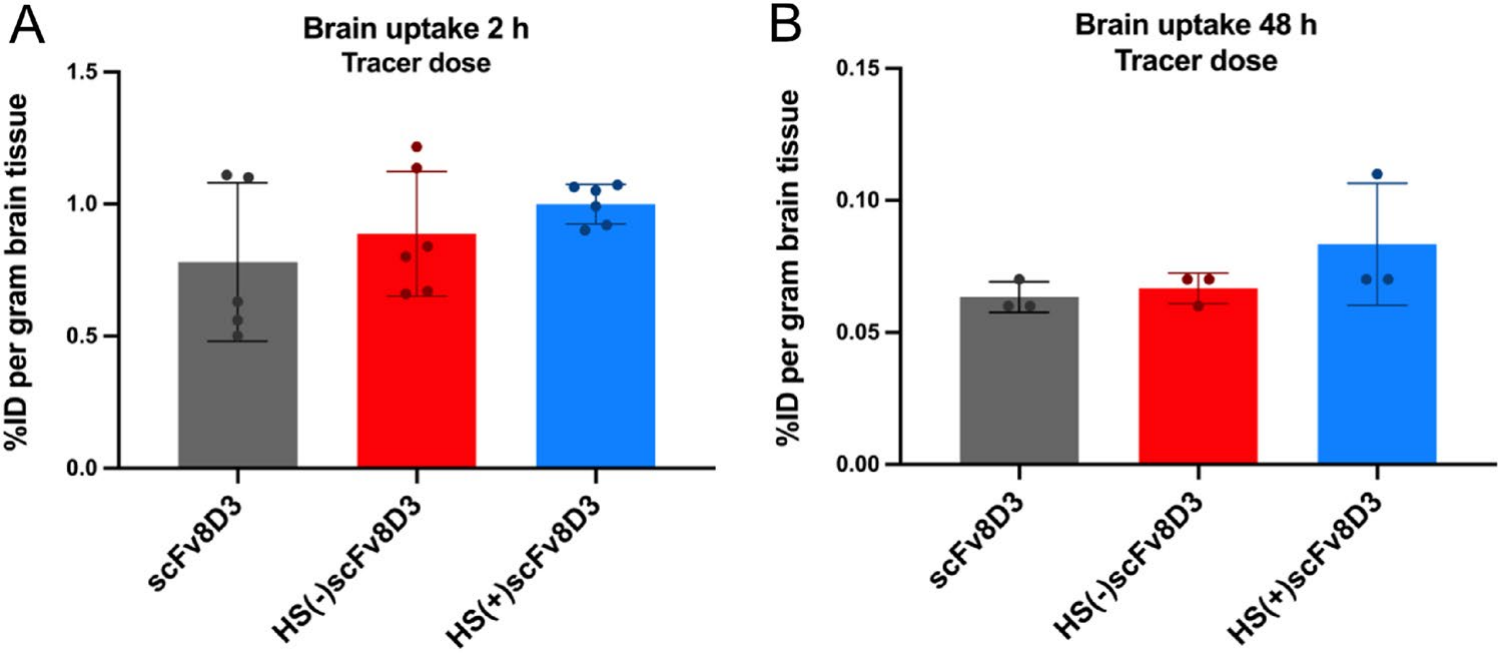
Brain uptake of tracer doses of [^125^I]scFv constructs. (**A**) The brain uptake of the three [^125^I]scFvs at 2 h post injection. [^125^I]scFv8D3 (n = 5), [^125^I]HS(−)scFv8D3 (n = 6) and [^125^I]HS(+)scFv8D3 (n = 6). (**B**) The brain uptake of a tracer dose of the three [^125^I]scFvs at 48 h post injection. [^125^I]scFv8D3 (n = 3), [^125^I]HS(−)scFv8D3 (n = 3) and [^125^I]HS(+)scFv8D3 (n = 3). Results are presented as mean ± SD.

After confirming that the HS(+)scFv8D3 mutant binds stronger to HS than HS(−)scFv8D3 and scFv8D3 (Fig. 4), we performed in vivo brain uptake experiments to determine whether HS-binding affects the ability of scFv8D3 to traverse the BBB. The constructs were labelled with ^125^I and a tracer dose of 0.3 nmol/kg was administered intravenously. The levels of [^125^I]scFv8D3, [^125^I]HS(+)scFv8D3 and[^125^I]HS(−)scFv8D3 were comparable for all three constructs 2-h (Fig. 6A) and 48 h (Fig. 6B) post-injection. The mean brain uptake 2 h post injection was 0.78% of measured radioactivity per gram of tissue divided by total injected radioactivity (%ID/g) for scFv8D3, 0.89%ID/g for the HS(−)scFv8D3 mutant and 0.99%ID/g for the HS(+)scFv8D3 mutant (Fig. 6A). The mean brain uptake at 48 h post-injection was 0.06%ID/g for scFv8D3, 0.07%ID/g for the HS(−)scFv8D3 mutant and 0.08%ID/g for the HS(+)scFv8D3 mutant (Fig. 6B).

### Brain uptake of therapeutic dose of scFv8D3 and HS mutants

**Figure 7:**
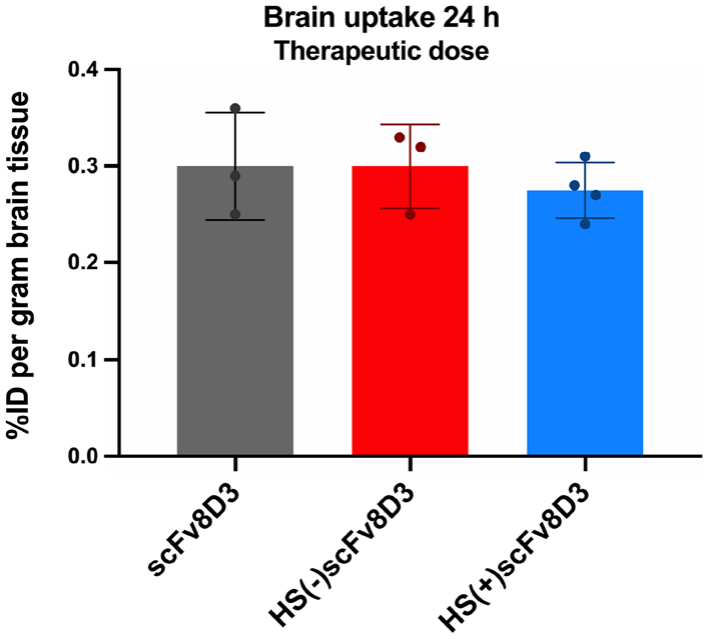
Brain uptake of therapeutic doses of [^125^I]scFvs constructs. Brain uptake values for the three [^125^I] labelled scFv constructs administered intravenously at a therapeutic dose (30 nmol/kg). The activity was measured 24 h post-injection. [^125^I]scFv8D3 (n = 3), [^125^I]HS(−)scFv8D3 (n = 3) and [^125^I]HS(+)scFv8D3 (n = 4). Results are presented as mean ± SD.

Next, we wanted to investigate if the three scFvs constructs had different brain uptake values at a therapeutic dose. It has been shown previously that in vivo brain uptake of high affinity TfR binders is increased at tracer doses compared to high doses^21,45^. Being that all scFvs had a similar affinity to TfR (Fig. 3B) we wanted to see if introducing or removing HS binding sites in the scFv8D3 had an effect on brain uptake at higher administered doses. We tested the constructs at a therapeutic dose of 30 nmol/kg, which is 100-fold higher than the tracer dose previously used. The brain uptake was similar for all three [^125^I]scFvs under these conditions, with the observed mean brain uptakes of 0.30%ID/g for scFv8D3, 0.30%ID/g for HS(−)scFv8D3 and 0.28%ID/g for HS(+)scFv8D3, 24 h post-injection (Fig. 7).

### Investigation of brain parenchymal vs brain capillary distribution of scFv8D3 and HS mutants

**Figure 8:**
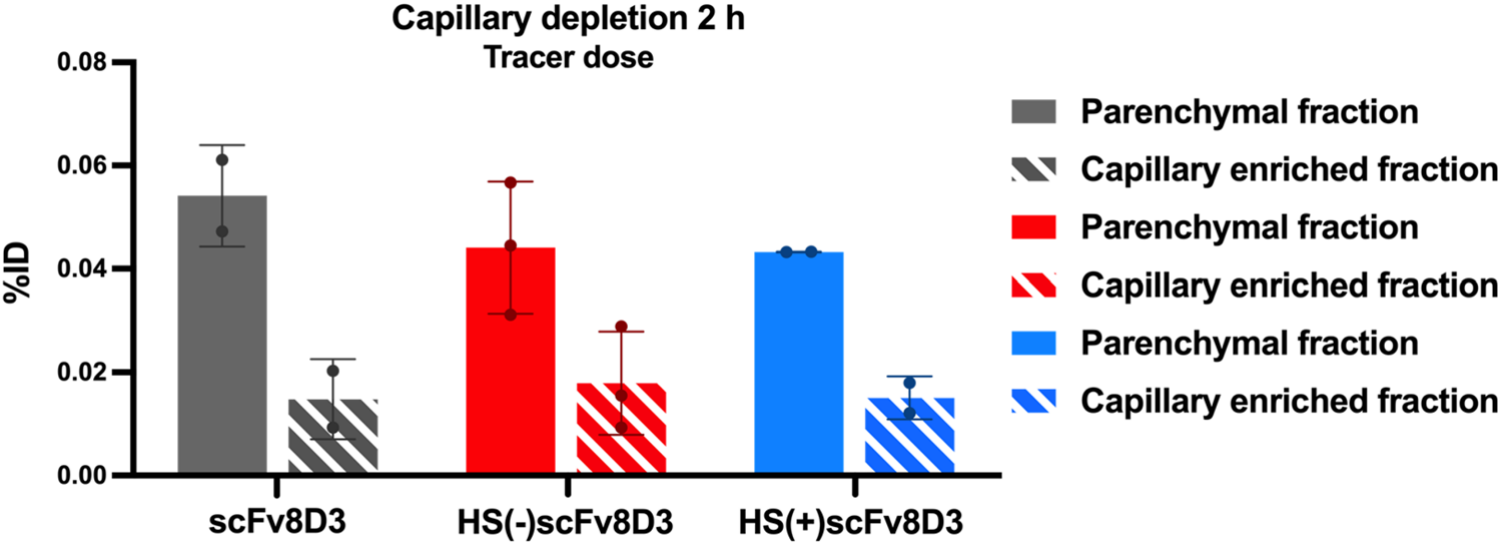
Signal distribution in brain parenchyma vs brain capillaries of [^125^I]scFv constructs by capillary depletion. The CD results were attained from ex-vivo studies 2 h post injection of WT animals intravenously injected with tracer doses (0.3 nmol/kg) of [^125^I]scFv8D3 (n = 2), the [^125^I]HS(−)scFv8D3 mutant (n = 3) or the [^125^I]HS(+)scFv8D3 mutant (n = 2). The CD results are presented as average radioactivity normalized to the injected dose (%ID) in MBq.

The brain uptake results that we obtained by measuring the radioactivity of the radiolabeled [^125^I]scFvs ex-vivo at different dosages indicated that HS binding did not affect the brain penetrance of scFv8D3. However, the method of measuring brain uptake does not distinguish between radioactive signal inside the brain parenchyma and signal in, or at the cell surface of, the BECs of the BBB. To overcome this, we used qualitative capillary depletion (CD)^46,47^ and semi-quantitative nuclear track emulsion (NTE) methodologies^48^, which are methods capable of differentiating the radioactive signal in brain parenchyma and BECs of the BBB. For the CD and NTE ex vivo experiments, the [^125^I]scFv constructs were intravenously administered at tracer doses (0.3 nmol/kg), after which the animals were sacrificed at 2- h post injection. The CD ex vivo experiment showed that the three [^125^]scFvs were present in both the brain parenchymal and capillary enriched fractions, with approximately 75% of the signal present in the brain parenchyma enriched fraction (Fig. 8). These results are consistent with a previously studied fusion protein containing scFv8D3^48^.

**Figure 9:**
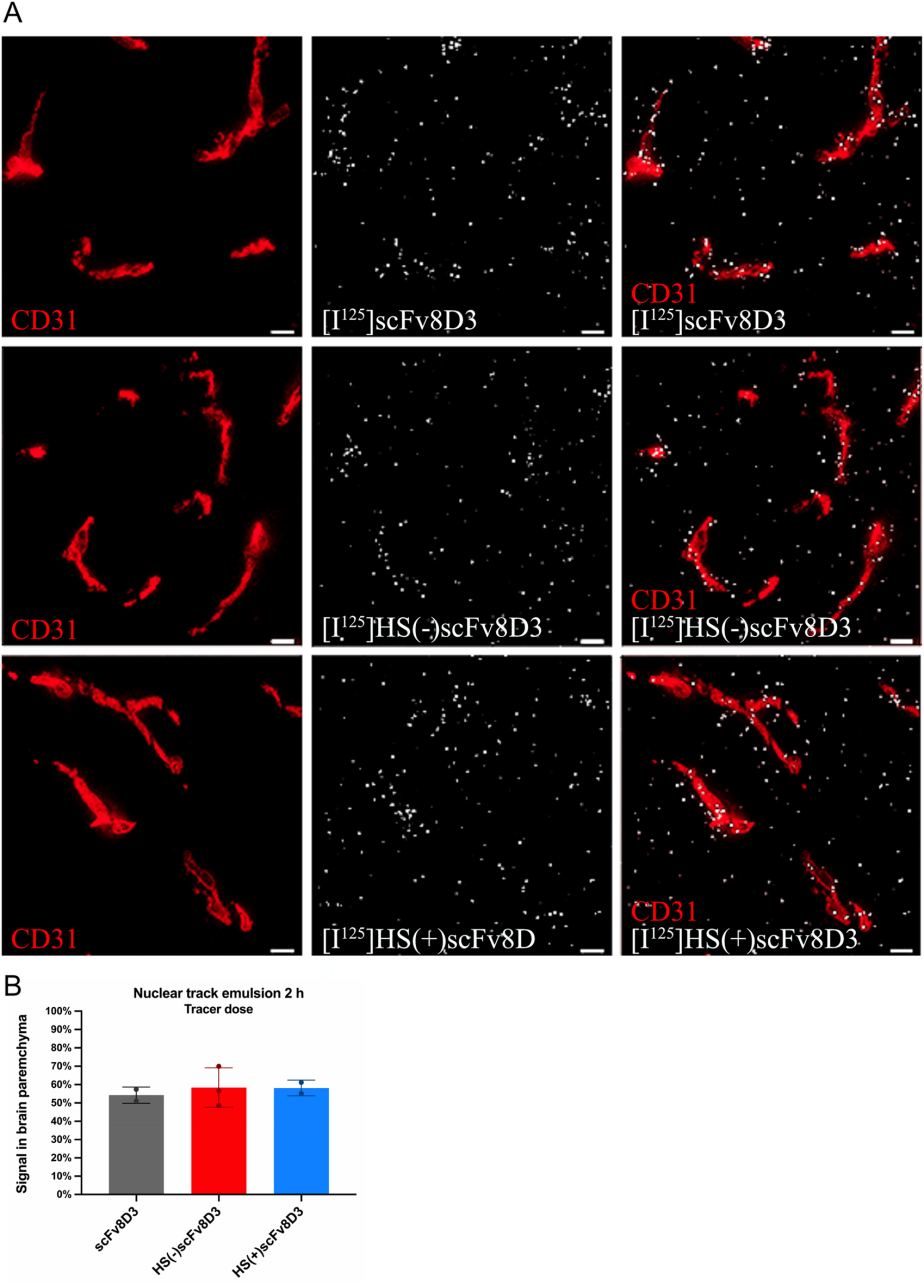
Ex-vivo brain nuclear track emulsion (NTE) shows the three [^125^I]scFv constructs similarly distributed in the brain parenchyma. The NTE results were attained from ex-vivo 2 h post injection studies of animals injected with tracer dose (0.3 nmol/kg) of [^125^I]scFv8D3 (n = 2), the [^125^I]HS(−)scFv8D3 mutant (n = 3) or [^125^I]HS(+)scFv8D3 mutant (n = 2). (**A**) Representative images of NTE (white puncta) detecting [^125^I]scFv8D3, [^125^I]HS(−)scFv8D3 mutant or [^125^I]HS(+)scFv8D3 mutant, and CD31 brain capillary staining (red) in mouse brains. White scale bar = 10 μm. (**B**) Semi-quantification of NTE images was performed for the association of white puncta (antibody-derived signal) within the brain parenchyma or colocalized with the CD31-fluorescently stained brain capillaries. Results are presented as mean ± SD.

The nuclear track emulsion data resembled the findings of the CD by confirming the presence of all three [^125^I]scFvs constructs in the brain parenchyma (Fig. 9A). Semi-quantitative analysis demonstrated 54% of the signal in the brain parenchyma for [^125^I]scFv8D3 and 58% for [^125^I]HS(−)scFv8D3 and [^125^I]HS(+)scFv8D3 (Fig. 9B).

### The HS plus mutant is not present in urine in contrast to the HS minus mutant and scFv8D3

**Figure 10:**
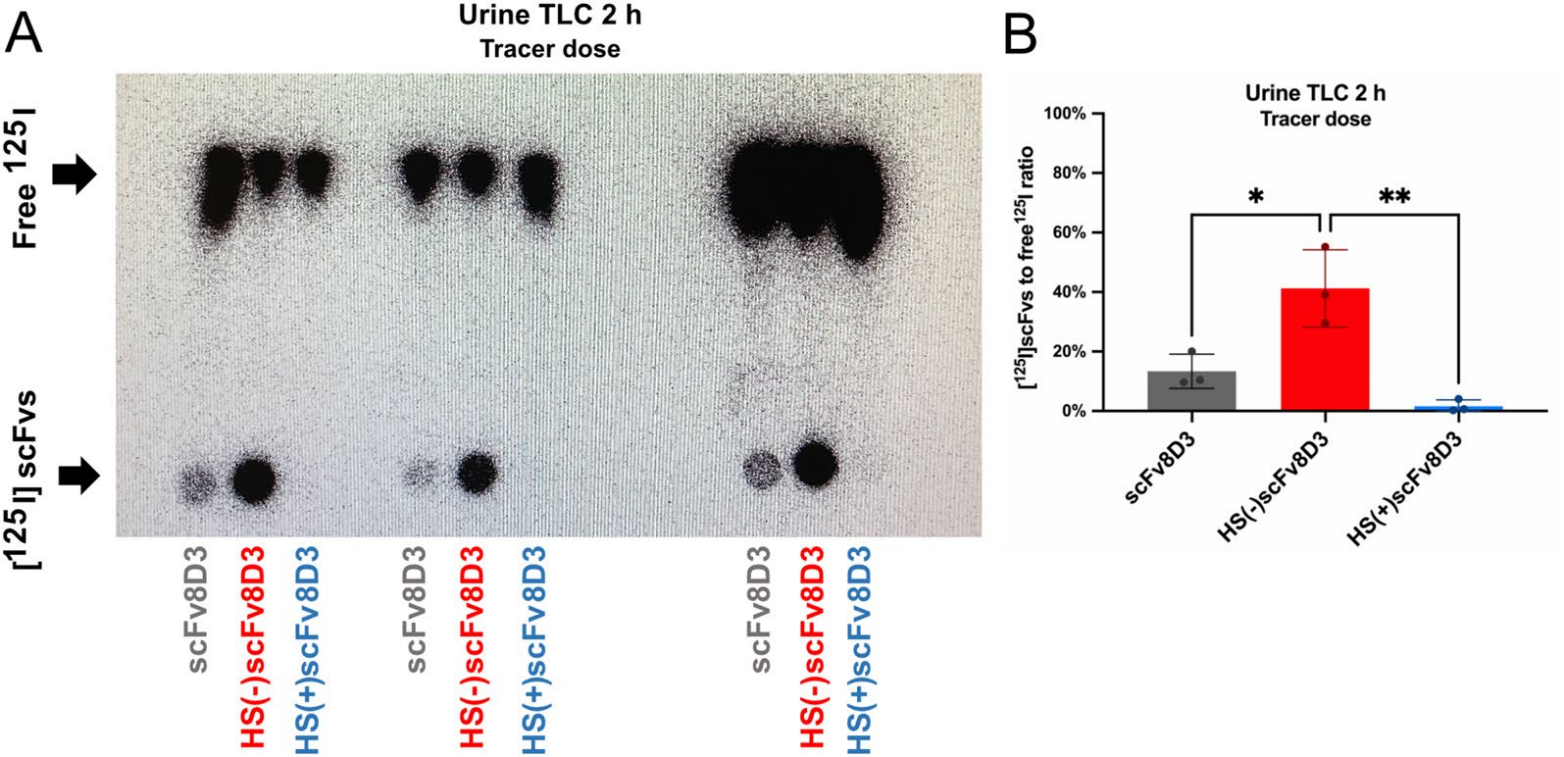
Urine Thin-Layer Chromatography quality controls of [^125^I]scFvs constructs. (**A**) The urine TLC quality control of the radiolabeled scFvs [^125^I]scFv8D3, (n = 3), [^125^I]HS(−)scFv8D3 (n = 3) and [^125^I]HS(+)scFv8D3 (n = 3) 2 h post-injection. (**B**) Semi-quantitative analysis of the ratio between the [^125^I]scFvs to free ^125^I by measuring optical density of the TLC signal with Fiji (ImageJ)^44^. Results are presented as mean ± SD and statistical comparisons were conducted between the three [^125^I]scFvs. * Represents a significance of P < 0.05, ** Represents a significance level of P < 0.01. Full sized image of TLC membrane in supplementary Fig. S2. Low exposure image of TLC membrane in supplementary Fig. S3.

Urine from the animals in the ex vivo studies was analyzed with TLC to investigate renal elimination of the radiolabeled scFvs, as their size (approximately 31 kDa) is below the glomerular filtration cut off (approximately 50 kDa)^49^. In contrast to the brain uptake of the [^125^I]scFvs, we did see a clear difference in the presence of the three [^125^I]scFvs in the urine from the animals. The [^125^I]HS(+)scFv8D3 mutant is almost undetectable in the urine and [^125^I]scFv8D3 is detected at a low level. Conversely, the [^125^I]HS(−)scFv8D3 mutant gives a strong signal (Fig. 10A) 2 h post-injection of a tracer dose (0.3 nmol/kg). Full sized, and low exposure, images of the TLC membrane are shown in supplementary Figs. S2 and S3 respectively. The ratio of signal from [^125^I]scFv to free ^125^I is significantly higher for the [^125^I]HS(−)scFv8D3 mutant compared to [^125^I]scFv8D3 and [^125^I]HS(+)scFv8D3 in urine 2 h post-injection (Fig. 10B).

### Free iodine-125 brain penetrance and blood distribution

**Figure 11:**
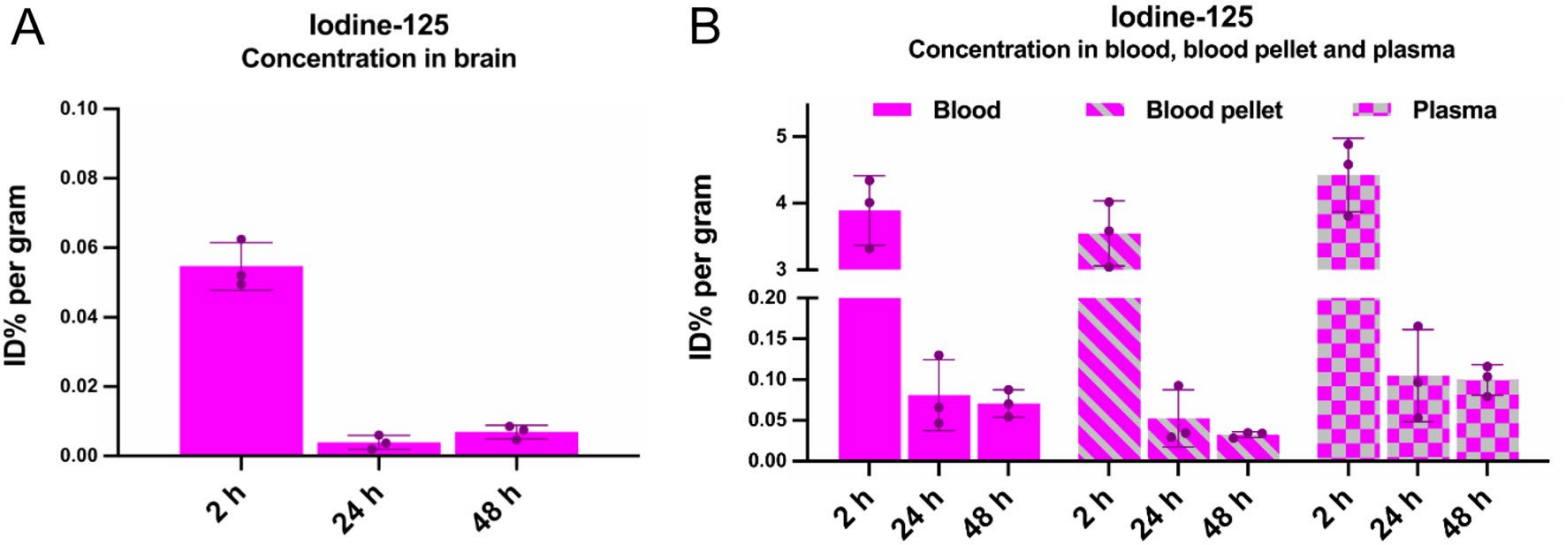
Brain penetrance and blood distribution of iodine-125. The brain penetrance and blood distribution of ^125^I was investigated ex-vivo by measuring the activity 2 h (n = 3), 24 h (n = 3) and 48 h (n = 3) post intravenous injection in WT-mice, presented as of %ID/g. (**A**) Ex-vivo concentration of ^125^I in brain. (**B**) Blood distribution of ^125^I. Results are presented as mean ± SD.

Discovering the fast in vivo degradation of the three [^125^I]scFvs, and the high degree of free ^125^I present in the plasma (Fig. 5), the brain penetrance of free ^125^I was investigated to confirm that the radioactive signal measured in the ex-vivo brain uptake experiments was [^125^I]scFvs. ^125^I was intravenously administered and the brain concentration, as well as blood distribution, was measured at 2-, 24- and 48 h post injection. At the 2 h time point, the mean brain concentration of ^125^I was 0.05% %ID/g (Fig. 11A), with a plasma concentration of 4.4%ID/g (Fig. 11B). At the 24 h time point, the mean brain concentration of ^125^I was 0.004%ID/g (Fig. 11A), with a plasma concentration of 0.11%ID/g (Fig. 11B). At the 48 h time point the mean brain concentration of ^125^I was 0.007%ID/g (Fig. 11A), with a plasma concentration of 0.10%ID/g (Fig. 11B).

## Discussion

The aim of this study was to investigate if HS binding of TfR-binding BBB transporters affects their brain uptake. We were successful in creating new HS binding cluster sites as we could substantially increase the binding of the HS(+)scFv8D3 mutant to heparin in vitro*,* demonstrating that it is possible to add HS binding clusters by point-mutations based on the design and approach presented here.

The HS binding capacity of the three scFvs were analyzed with heparin LC. Although heparin and HS are structurally different molecules, both consist of sulfated glycosaminglycan chains, and ligands that can bind heparin can also bind HS ^31,36,50–52^. Importantly, it has been shown that transferrin binds both heparin and HS^36,51^. The HS(+)scFv8D3 mutant was able to bind the heparin column and was eluted in two peaks. One possible explanation as to why HS(+)scFv8D3 elutes in two peaks might be differentially protonated histidines. It has been shown that the pKa of histidine residues are affected by the degree of hydrophobicity of their environment in folded proteins^53^. Furthermore, it has been previously reported that a monoclonal antibody eluted as two distinct peaks during a linear salt gradient due to differential histidine protonation^54^. Alternatively, the second peak could be HS(+)scFv8D3-multimers, which through the avidity effect would have more HS binding clusters and motifs interacting with the column. In contrast, the HS(−)scFv8D3 and scFv8D3 were not able to bind the heparin column and showed no elution peaks. Surprisingly, scFv8D3 did not exhibit binding to the heparin column even though there are predicted HS binding sites present in structure homology model. Even though there are possible HS binding clusters and motifs in scFv8D3, they may not bind strongly enough to the column under the conditions of the experiment. Furthermore, the clusters and motifs that we observed in the model are not necessarily accurate in regard to the angular orientation of the R-groups of the positively charged residues, meaning that the R-groups might not be at the correct distances from each other. An alternative explanation is that the HS binding sites that we found using the structure homology model are not present in the real structure of scFv8D3. However, due to the fact that the model allowed us to successfully introduce new HS binding clusters in the HS(+)scFv8D3 mutant, we believe that this explanation is less likely.

The point mutations that were introduced did not affect the TfR binding capacity of the scFv8D3 mutants, which suggests that the mutations were not detrimental for the function of the scFvs. To avoid affecting scFv8D3s TfR binding capacity, amino acids present within the variable regions of scFv8D3 were not mutated. Introducing the point mutations in the variable regions of scFv8D3 would not necessarily have affected its TfR binding affinity, however, if the TfR binding affinity would have been altered it would have been hard to determine whether any difference in brain uptake was the result of HS binding capacity or due to the change of TfR affinity. To meet this constraint, and with criterion of only adding HS binding clusters at the surface of the protein, mutations were only introduced to the side of the scFv that was opposite to the TfR binding domain. In relation to receptor-mediated endocytosis, the benefit of having HS binding clusters or motifs near the receptor binding domain (or if HS-binding should be simultaneous to or before receptor binding), is unknown. When analyzing the surface of transferrin, we could identify many potential HS binding clusters and motifs, out of which only one is within the TfR binding domain. If simultaneous HS and receptor binding is beneficial in this context, it would follow that adding the HS binding site outside of the receptor binding domain is the preferred option.

Based on our results, it seems like scFv8D3 is not dependent on the HS binding clusters that we identified for its brain uptake. Moreover, our results indicate that the HS binding cluster that we introduced to the HS(+)scFv8D3 mutant does not improve its brain uptake, with similar levels determined for all three [I^125^]scFvs in all ex-vivo studies conducted. The brain uptake results were confirmed by CD and NTE, which demonstrates that the increased number of HS binding sites present in HS(+)scFv8D3 does not lead to increased binding to the brain capillaries, but rather the brain parenchymal uptake was similar to that of scFv8D3 and HS(−)scFv8D3. Additionally, the distribution between brain parenchyma and brain capillaries of the three [I^125^]scFvs were similar to another scFv8D3-containing BBB shuttle of similar size which has been previously studied^48^. One potential explanation for the lack of increased brain uptake of HS(+)scFv8D3 is that even though it might be unable to bind some HS patterns it might still be unable to the HS present at the BBB, due to the fact that HS patterns are heterogenous with varying tissue composition^27–29^.

All three [I^125^]scFv constructs were degraded rather quickly in vivo, with the [I^125^]HS(+)scFv8D3 mutant demonstrating a particularly rapid degradation, based upon the high ratio of free I^125^ to [I^125^]scFvs at the 3- and 6 h time points (Fig. 5). The radiolabeling of the scFvs was done with I^125^ (tyrosine iodination), which has high labeling stability in regard to non-enzymatic deiodination^55,56^. However, it has been shown that in vivo deiodination of tyrosine radioiodinated proteins/peptides predominately occurs after proteolytic degradation of the radiolabeled proteins/peptides^55,57,58^. Due to the fast in vivo degradation of the three [I^125^]scFv constructs, and high free ^125^I concentration in plasma, the brain penetrance of free ^125^I was investigated as a control for our ex vivo brain uptake experiments. The brain penetrance of free ^125^I was expected to be low as the BBB prevents the diffusion of most substances^59^. Previous studies have shown that iodine has low blood-to-brain permeability, while it also diffuses freely from brain to cerebrospinal fluid (CSF), and is actively pumped out of the CSF^60^. Free iodine in blood circulation is mainly absorbed by the thyroid^61^, where it is actively taken up by transporter proteins and fixated in the form of thyroid hormones^62^. Iodine is also readily excreted via urine^63^. Furthermore, the delivery of ^125^I to brain tumors has been shown to be significantly increased by the use of BBB-disruption^64^, indicating that the BBB limits the brain penetrance of free I^125^. The brain penetrance of free I^125^ was indeed found to be low compared to the brain uptake of the [I^125^]scFvs. In order to determine whether the free I^125^ signal might contribute to our [I^125^]scFvs brain uptake results, the contribution of free I^125^ was estimated and found to be minuscule (Tables S1-, S2- and S3), leading to the confirmation of our initial interpretations.

It has been shown previously that cationization (increasing the net positive charge and/or decreasing the net negative charge) of molecules can increase their brain uptake^65^. It is postulated that this is due to adsorptive-mediated endocytosis (AME), where an increased positive charge of the molecule facilitates interaction with the net negatively charged BCEC membranes, that have a high proportion of HS binding sites^66–69^. This strategy has been applied to increase the brain uptake of cationized antibody fragments (Fabs)^70,71^. However, even though the HS(+)scFv8D3 mutant contained more positively charged amino acids compared to scFv8D3, and thus had an increased positive net charge, the brain uptake was not affected. This might be due to the relatively low amount of cationization of HS(+)scFv8D3 since only two amino acids were mutated and thus the positive charge increase was low compared to cationization performed on Fabs^70,71^. Due to the low in vivo stability of the three [I^125^]scFvs, the radioactivity measured in the peripheral organs consists of both [I^125^]scFvs and free ^125^I, as well as partly degraded scFvs. Nevertheless, the peripheral and blood distribution was similar in general for all three scFvs at both tracer and therapeutic doses spanning all time points measured (Figs. S2–5).

Interestingly, there was a significantly reduced level of [I^125^]HS(+)scFv8D3 and [I^125^]scFv8D3 in the urine 2 h post-injection, compared to [I^125^]HS(−)scFv8D. Confoundedly, these results do not correlate with the levels found in the plasma at 3- and 6 h post-injection. While the [I^125^]HS(+)scFv8D3 mutant was significantly reduced in the plasma compared to [I^125^]scFv8D3, [I^125^]HS(−)scFv8D3 was the most prominently present [I^125^]scFv in urine while being similarly present in plasma compared to [I^125^]scFv8D3. The difference in plasma and urine can be speculatively interpreted as indicative of decreased renal elimination and increased renal catabolism of the [I^125^]HS(+)scFv8D3 mutant, due to increased renal tubular reuptake. While the increased renal elimination of the [I^125^]HS(−)scFv8D3 mutant might be due to decreased renal reuptake. The molecular weight (31 kDa) of the scFvs is not high enough to exclude renal elimination. The current literature suggests that molecules weighing less than 50 kDa^49^ can pass through fenestrations of the kidney glomeruli^72,73^. The pore size of the kidney glomeruli is approximately 8 nm^72^, which limits the rate of clearance of protein-based drugs depending on their size, where smaller proteins are able to be cleared to a larger extent^49^. It has been shown that the renal elimination of small proteins (< 50 kDa), which can pass through the glomerular membrane, is decreased in patients with impaired tubular reuptake due to tubular pathology resulting in tubular proteinuria of small proteins^74^. HSPGs are abundantly present in endothelial cells membranes of the kidneys^75,76^ and since HSPG alterations in the kidney tubules has been implicated in tubular proteinuria of small proteins^77^ we speculate that binding to the HS might decrease the renal clearance of the HS(+)scFv8D3 mutant due to increased reabsorption. A likely fate of the HS(+)scFv8D3 mutant after reabsorption by the kidney tubules is catabolization by proteolytic degradation, as the renal tubules are the main site for catabolization of low molecular weight (10–50 kDa) plasma proteins^78–83^. Other possible fates of the HS(+)scFv8D3 mutant is HSPG-mediated endocytosis and subsequent lysosomal degradation in capillary endothelial cells or hepatocytes, as both cell types have been reported to degrade proteins present in plasma^84^. If HS(+)scFv8D3 had been released back intact into the bloodstream after being endocytosed its plasma half-life should have been prolonged, which we did not observe. The current knowledge about HSPGs role in renal clearance of large proteins (> 50 kDa) suggests that they are necessary for maintaining the glomeruli pore sizes and fenestration of the endothelium^49,72,73,85^. Conversely, a study has shown that the small (24 kDa) protein Azurocidin, which is also called heparin binding protein, is renally cleared to a low extent in healthy individuals despite its small size, which might be due to it binding HSPGs in the kidney tubules^86^. In light of this, we can speculate that scFv8D3 binds to HS to a sufficient extent to decrease its renal elimination. To the best of our knowledge, there are no previous studies where the same protein, with either increased or decreased number of HS binding sites, has been investigated. The findings of our study indicate that HS binding is relevant to the renal elimination of scFvs and possibly other low molecular weight proteins. One potential application of our findings is that the addition of HS binding sites might enable the delivery of low molecular weight protein drugs to the renal tubules, enabling studies into the renal reabsorption of proteins.

In summary, we did not find any evidence supporting our hypothesis that the HS binding clusters that we introduced to the TfR binder scFv8D3 increases its brain uptake, or that the brain uptake of scFv8D3 is dependent on the HS binding sites that we identified. From this we can consider it likely that HS binding is not a property that is of importance for the brain uptake of the scFv8D3 brain shuttle. In contrast, the low signal detected for the HS(+)scFv8D3 mutant in urine could be interpreted as indicative of decreased renal elimination. We speculate that the decrease of renal elimination could be due to HS(+)scFv8D3 binding to HS at the kidney tubular endothelium, which in turn may increase its renal reabsorption, and subsequent degradation. The introduction of HS binding sites to small (≤ 50 kDa) protein-based protein drugs might be beneficial for targeting the renal tubular endothelium.

## Methods

### Design of a HS-binding TfR binding BBB-transporter

The scFv8D3BBB transporter (31 kDa Mw)^13,15^ consists of the heavy and light chain variable domains of the TfR binding antibody 8D3^16^ connected to each other by a linker consisting of the amino acids SGSTS (G4S)_3_^87^. The scFv8D3 and the two new mutant constructs were designed with a twin strep tag (WSHPQFEKGGGSGGGSGGSAWSHPQFEK), which was linked to the C-terminal with a TEV linker (ENLYFQS) followed by an in-house designed linker (APGSGTGSAPG). The constructs were cloned into the expression vector pcDNA3.4 (GeneArt, Thermo Fisher) with our standard signal peptide added to the N-terminal. The structure of scFv8D3 was generated by using the automated protein structure homology-modelling server SWISS-MODEL^88^ based on the primary amino acid structure of scFv8D3. The generated structure was analyzed in the protein structure software PyMOL^89^ and HS binding motifs were searched for on the surface of the protein. There were no suitable regions available on the surface of scFv8D3 for the creation of linear HS binding motifs. Instead, two positions in folded regions of scFv8D3 with two adjacent positively charged amino acids were found, allowing for the creation of HS binding motifs by introducing a third positively charged amino acid by point mutation at the correct distance of ~ 5–10 Å, which mimics a distance of one or two amino acid sin the linear HS binding motifs BB-X-B or BB-XX-B^30,31^. Amino acid present within the variable regions of scFv8D3 were not mutated, to avoide affecting the TfR-binding affinity. Additionally, to prevent introducing protein instability, mutating highly hydrophobic amino acids into basic amino acids, and vice versa, was avoided since hydrophobicity is one of the forces affecting protein folding^41,42^. Two HS binding motifs were added to scFv8D3 by point mutations, S75K and P176K, creating the HS(+)scFv8D3 mutant. Two potential HS binding motifs at the surface of scFv8D3 were removed by point mutations, K42Q, K43Q, K243Q and R244Q, creating the HS(−)scFv8D3 mutant.

### Recombinant expression and purification

All three scFvs were produced as previously described^90^. In short, human Expi293 cells were transfected with the pcDNA 3.4 vectors containing the constructs by using the transfection reagent polyethylenimine (PEI) (Polysciences 24765-1) and the cell-cycle arrester valproic acid (VPA) (Sigma P4543). Seven days’ post-transfection, the cells were harvested and the supernatant was separated by using Celpure^®^ P300 (Sigma-Aldrich) and filtered through low protein binding 0.22 μm filter units (Corning 430513). The scFvs were purified with an Äkta start liquid chromatography (LC) instrument with Strep-Tactin^®^XT 4Flow^®^ cartridge (IBA Life Sciences 2-5021-001). The scFvs were eluted with a 50 mM biotin- Tris HCl buffer and then immediately concentrated by Amicon centrifugal filters (Sigma-Aldrich, UFC501024) and buffer exchanged to PBS (Gibco) with Zeba spin columns (ThermoFisher, A44301). The concentrations of the purified proteins were determined by measuring their absorbance at 280 nm (nm) and calculating their concentrations by factoring in their molecular extinction coefficients and Mw.

### SDS-PAGE and purity analysis

To confirm the size and purity, the purified scFvs were analyzed using SDS-PAGE followed by coomassie blue staining. For the SDS-PAGE, the scFvs were loaded together with NuPAGE™ LDS Sample Buffer (Invitrogen™) and a pre-stained protein marker (ThermoFisher 26616) on NuPAGE™ Bis–Tris 4 to 12% 15-well precasted gels (Invitrogen™) without adding reducing agents, and run at 80 V for approximately 1.5 h in MES running buffer (NuPAGE™ MES SDS Running Buffer, Thermo Fisher). The gels were then stained with comassie blue staining (PageBlue™ Protein Staining Solution, Thermo Scientific™) for 20 min, rinsed with deionized water and photographed using Image Studio software (version 5.2.5). The acquired image was analyzed with Fiji(ImageJ).

### Transferrin receptor ELISA

The TfR binding ability of the scFvs was assessed by a previously described TfR ELISA^91^. Ninety-six wells half area plates (Corning Incorporated) were coated with 50 ng/well recombinant mouse TfR extracellular domain protein in PBS (prepared in our lab) and stored overnight at 4 °C. The following day, the plates were blocked with 1% BSA in PBS for two hours at room temperature (RT) while shaking and then incubated with serial dilutions of the scFvs for two hours at RT while shaking. For detection, a mouse-anti Strep-Tag II (IBA Life Sciences) primary antibody and an HRP-conjugated goat anti-mouse secondary antibody (Sigma-Aldrich 12-349) were used. The signal development was done with K-blue aqueous TMB (Neogen Corp) and the absorbance was measured using FLUOstar Omega microplate reader (BMG Labtech) at 450 nm. The scFvs and antibody dilutions were made in ELISA incubation buffer (1× PBS with 0.1% BSA and 0.05% Tween-20). Washing steps were made done with ELISA washing buffer (1× PBS with 0.05% Tween-20) five times.

### Thermal shift assay

The stability of the scFvs was investigated by Tycho nt.6 instrument (NanoTemper technologies, München, Germany) as previously described^43^. In short, 8 µl of each scFv at 1.2 µM concentration were heated in glass capillaries with fluorescence intensities measured at 330 nm and 350 nm during a linear temperature increase from 35 to 95 °C.

### Heparin column liquid chromatography

The heparan sulfate binding capacity of the scFvs was assessed by heparin column liquid chromatography with an Äkta start instrument. Prior to loading the scFvs onto the HiTrap Heparin HP 1 mL column (Cytiva), the proteins were buffer exchanged from PBS to 10 mM sodium phosphate buffer (pH 7) using Zeba™ Spin Desalting, 7KColumns (ThermoFisher Scientific) to prevent disruption of binding to the heparin column caused by the salts in the PBS buffer. The scFvs were loaded at a rate of 0.5 mL/min. After loading of the scFvs, the column was washed with 10 mL of binding buffer and the wash was collected. The elution (measured by monitoring absorbance at 280 nM) was performed with a linear gradient of elution buffer (measured by conductivity, mS/cm) over 15 mL of 0% to 100% elution buffer (0.5 M NaCl in 10 mM sodium phosphate buffer) at a rate of 1 mL/min and collected.

After elution, the fractions were analyzed by SDS-PAGE followed by Coomassie staining.

### Animals

For all animal experiments, 3–4 months old (C57BL/6JBomTac, ordered from Taconic) wild-type male and female mice were used. The animals were housed in individually ventilated cages (4–5 animals/cage) in rooms with controlled temperature (20–22 °C) and controlled humidity (50–55%). The mice were feed ad libitum with free access to water and had daily surveillance by trained personnel in an animal facility at Uppsala University. All procedures described in this paper were approved by the Uppsala County Animal Ethics board 5.8.18-20401/20 and performed according to the ARRIVE guide lines. The rules of and regulations of the Swedish Animal Welfare Agency, as well as the of European Communities Council Directive of 22 September 2010 (2010/63/E.U.), were followed during the animal studies and all efforts were made to minimize animal suffering and to reduce the number of animals used.

### Radiolabeling with iodine-125

The scFvs variants were labelled with iodine-125 (^125^I) using Chloramine-T as described previously^92^. In short, 400 nM of the three scFvs were mixed with ^125^I stock solution (Perkin Elmer Inc, Waltham) and 1 mg/mL of Chloramine-T (Sigma Aldrich) in PBS. After 90 s of incubation, the reaction was stopped with 1 mg/mL sodium meta-bisulphite (Sigma 08982). The radio-labeled scFvs were then purified from unbound and free ^125^I by using NAP columns (VWR 17-0853-02) and eluted in PBS. The radiolabeling procedures were done 1–2 h before the ex vivo studies. The yield of the labeling process varied between the different studies, due to inherent variation of the method, however this was accounted for in the dose calculations. The yield was approximately 22.8 MBq/nmol for the in vivo 96 h plasma stability study, 35 MBq/nmol for the 2 h and 48 h ex vivo studies, approximately 12 MBq/nmol for the NTE/CD ex vivo study (also 2 h) and approximately 3.5 MBq/nmol for the 24 h therapeutic ex vivo study.

### In vivo 96 h plasma stability study

Plasma stability of scFv8D3, HS(+)scFv8D3 and HS(−)scFv8D3 mutant were investigated in C57Bl/6 wild-type mice (3–4 months old). The mice were injected with a tracer dose (0.3 nmol/kg, 0.04 mg/kg) of each scFv. Mice (total n = 15, 5 mice for each protein) were intravenously injected via the tail vein with 1.10 ± 0.04 MBq [^125^I]scFv8D3, 1.06 ± 0.06 MBq [^125^I]HS(−)scFv8D3 or 1.24 ± 0.10 MBq [^125^I]HS(+)scFv8D3. Approximately thirty microliters blood samples were collected were obtained from the tail vein at 3-, 6-, 24-, 48- and 96 h post-injection. The blood samples were then centrifuged at 15,000×*g* for 5 min to obtain plasma and blood pellet samples. The mice were euthanized at 96 h post-injection by transcardial perfusion with 0.9% physiological saline under terminal anesthesia. The brains were dissected and divided into two hemispheres. The right hemisphere was left intact while the left hemisphere was divided in cerebrum and cerebellum. Liver, spleen, heart, lung, kidney, pancreas, muscle, bone, skull, and thyroid were also isolated. The radioactivity in the brain, peripheral organs, blood and urine was measured using a γ-counter (1480 Wizard, Wallac Oy, Turku, Finland). Plasma and urine were analyzed using thin-layer chromatography (TLC) for analysing the ratio of [^125^I]-labelled scFvs vs free ^125^I. Briefly, the bottom of a glass jar was filled with 70% acetone. Urine samples (~ 2 μl) were applied at a baseline on a piece of silica coated aluminum plate and allowed to dry for approximately 5 min before adding the TLC-plate to the solvent-containing glass jar, ensuring the solvent line was below the sample-baseline. When the solvent front had migrated two-thirds the way up the TLC-plate, it was removed from the glass container, allowed to dry for 15 min and developed underneath an X-ray film for 48 h in complete darkness. The X-ray film was then measured using a Cyclone Phosphoimager and the images obtained analyzed by ImageJ^44^.

### Ex-vivo 2 h biodistribution study

Brain uptake and peripheral biodistribution of scFv8D3, HS(+)scFv8D3 and HS(−)scFv8D3were investigated in C57Bl/6 wild-type mice (3 months old). The mice were injected with a tracer dose (0.3 nmol/kg, 0.04 mg/kg) of each scFv. Mice (total n = 9, 3 mice for each protein) were intravenously injected via the tail vein with 1.71 ± 0.09 MBq [^125^I]scFv8D3, 1.55 ± 0.07 MBq [^125^I]HS(−)scFv8D3 or 1.72 ± 0.05 [^125^I]HS(+)scFv8D3. Eight-microliter blood samples from the tail vein were collected at at 0.5-, 1-, and 2 h post injection. Euthanasia of the animals and dissection of the animals, radioactivity measurement, TLC quality control and data presentation was done in the same manner as described above in the 96 h in vivo plasma stability study.

### Ex-vivo 48 h biodistribution study

Half-life, brain uptake and peripheral biodistribution of scFv8D3, HS(+)scFv8D3 and HS(−)scFv8D3 was investigated in C57Bl/6 wild-type mice (3 months old). The mice were injected with a tracer dose (0.3 nmol/kg) of each scFv. Mice (n = 9, 3 mice for each protein) were intravenously injected via the tail vein with 1.82 ± 0.05 MBq[^125^I]scFv8D3, 1.6 ± 0.16 MBq [^125^I]HS(−)scFv8D3 or 1.66 ± 0.03 [^125^I]HS(+)scFv8D3. Eight-microliter blood samples from the tail vein were collected at 0.5-, 1-, 2-, 4,-, 6-, 24- and 48 h post injection. Euthanasia of the animals was carried out 24 h post-injection. Dissection of the animals, radioactivity measurement, TLC quality control and data presentation was done in the same manner as described above in the 96 h in vivo plasma stability study.

### Ex-vivo 24 h biodistribution study

Half-life, brain uptake and peripheral biodistribution of scFv8D3, HS(+)scFv8D3 mutant and HS(−)scFv8D3 was investigated in C57Bl/6 wild-type mice (3 months old). The mice were injected with a therapeutic dose (30 nmol/kg) of each scFv. Mice (n = 9, 3 mice for each protein) were intravenously injected via the tail vein with 0.31 ± 0.07 MBq [^125^I]scFv8D3, 0.38 ± 0.05 MBq [^125^I]HS(−)scFv8D3 and 0.29 ± 0.07 MBq [^125^I]HS(+)scFv8D3. Eight-microliter blood samples from the tail vein were collected at 1-, 2-, 4-, 6-, 24- and 48 h post-injection. Euthanasia of the animals was carried out 24 h post-injection. Dissection of the animals, radioactivity measurement, TLC quality control and data presentation was done in the same manner as described above in the 96 h in vivo plasma stability study.

### Ex-vivo brain nuclear track emulsion (NTE) and capillary depletion study (CD)

Brain uptake and peripheral biodistribution of scFv8D3, HS(+)scFv8D3 and HS(−)scFv8D3 was investigated in C57Bl/6 wild-type mice (3 months-old). The mice were injected with a tracer dose (0.3 nmol/kg) of each scFv. Mice (n = 9, 3 mice for each protein) were intravenously injected via the tail vein with 0.49 ± 0.02 MBq [^125^I]scFv8D3, 0.62 ± 0.1 MBq [^125^I]HS(−)scFv8D3 or 0.62 ± 0.03 [^125^I]HS(+)scFv8D3. Eight-microliter blood samples from the tail vein were collected at 0.5-, 1-, and 2 h post-injection. Euthanasia of the animals was carried out 24 h post-injection. Radioactivity measurement, TLC quality control and data presentation were done in the same manner as described above in the 96 h in vivo plasma stability study. The brains were dissected and divided into two hemispheres. The right hemisphere was left intact, measured and subsequently sectioned in readiness for NTE. The midbrain and cerebellum were dissected from the left hemisphere and the cortex was used for brain CD.

The ex vivo brain CD was performed as described previously^48^. Briefly, the brain cortices were isolated immediately after the animals were sacrificed by transcardial perfusion and then weighed and homogenized in 0.8 mL of cold physiological buffer (10 mM HEPES, 141 mM NaCl, 4 mM KCl, 2.8 mM CaCl_2_, 1 mM MgSO_4_, 1 mM NaH_2_PO_4_, 10 mM d-glucose adjusted to pH 7.4) in an ice-cold Dunce homogenizer. Subsequentially, 0.8 mL of 30% Ficoll 400 (Sigma Aldrich) was added to the homogenate, which was then stroked one additional time in the Dunce homogenizer. The homogenate was transferred to a 15 mL Falcon tube and centrifuged at 5200×*g* for 20 min at 4 °C, resulting in a parenchymal supernatant and a capillary enriched pellet, which was carefully separated from the supernatant. The radioactivity of the parenchymal supernatant fraction and the capillary enriched pellet fraction was measured in a γ-counter (PerkinElmer. The radioactivity for each fraction was normalized to the injected dose (%ID) in MBq.

Before the ex vivo NTE and immunofluorescence CD31-staining was performed, the frozen right hemispheres were cryosectioned sagittally (20 μm) in a cryostat (CryoStar NX70, Thermo Scientific) and mounted on glass slides. CD31 staining was applied to visualize the capillaries in the brain as previously described^46–48^. The brain sections were fixed in ice-cold methanol for 10 min, washed in PBS and then blocked for 1 h in 5% Normal Goat Serum. Slides were then washed again in PBS followed by washing buffer (0.1% Tween-20 in PBS) for 5 min while shaking. Sections were then incubated with 1.25 μg/mL rat-anti-mouse CD31 (BD, #553370) in PBS overnight. The next day sections were washed in washing buffer and subsequently incubated with the secondary antibody goat-anti-rat Alexa 647 for 1 h at room temperature. The sections were washed and the NTE experiment was performed in darkness as previously described^48^. Briefly, ILFORD K5 emulsion (Oxford Instruments, Gometz la Ville, France) was prepared as a 50:50 emulsion in MQ-water under heating in a 40 °C water bath. Previously CD31-stained brain sections were immersed in the ILFORD K5 emulsion for 10 s and the left to air dry for 2 h before incubating for 5 weeks at 4 °C. Sections were developed according to the to the manufacturer’s instructions, dehydrated in an ethanol concentration gradient (70%, 95% and lastly 100%) and mounted with Pertex (Histolab). Finally, the NTE and CD31-stained sections were imaged with a Zeiss Observer Z.1 microscope (Carl Zeiss Microimaging GmbH, Jena, Germany) and subsequentially the obtain images were processed equally using the ZEN software. The NTE-signal associated with capillary and parenchymal regions was quantified as previously described^48^. In short, 10 images from 2 to 3 individuals per scFv were analyzed with Fiji (ImageJ) by a standardized macro, and subsequentially the macro-generated results were quality controlled by manual inspection.

### Free iodine-125 brain penetrance and blood distribution study

Nine C57Bl/6 wild-type mice (2–3 months old) were intravenously injected via the tail vein with 0.84 ± 0.09 MBq ^125^I in saline. Mice were euthanized at 2 h (n = 3), 24 h (n = 3) and 48 h (n = 3) post-injection by a terminal blood sample from the heart, followed by transcardial perfusion with 0.9% physiological saline. The brain, whole blood, plasma, blood cell pellet (remaining after separation of plasma by centrifugation of whole blood) and major peripheral organs were isolated and the radioactivity was measured as described above in the 96 h in vivo plasma stability study.

### Statistics

Data are presented as mean ± SD. The data was tested for normality (gaussian distribution) followed by One-way ANOVA statistical test for the HS(−)scFv8D3 and HS(+)scFv8D3 mutants compared to the scFv8D3 for p-values: (*) < 0.05, (**) < 0.01, and (***) < 0.001.

## Supplementary Information

Supplementary Information.

## Data Availability

The datasets used and/or analyzed during the current study available from the corresponding author on reasonable request.
