# Supplementary material for: Introducing or removing heparan sulfate binding sites does not alter brain uptake of the blood–brain barrier shuttle scFv8D3
Source: Sci Rep. 2022 Dec 12;12:21479. doi: 10.1038/s41598-022-25965-x (PMC9744743; doi:10.1038/s41598-022-25965-x)
Supplement: Supplementary file 1 — Supplementary Information. [file 41598_2022_25965_MOESM1_ESM.docx]

Supplementary material


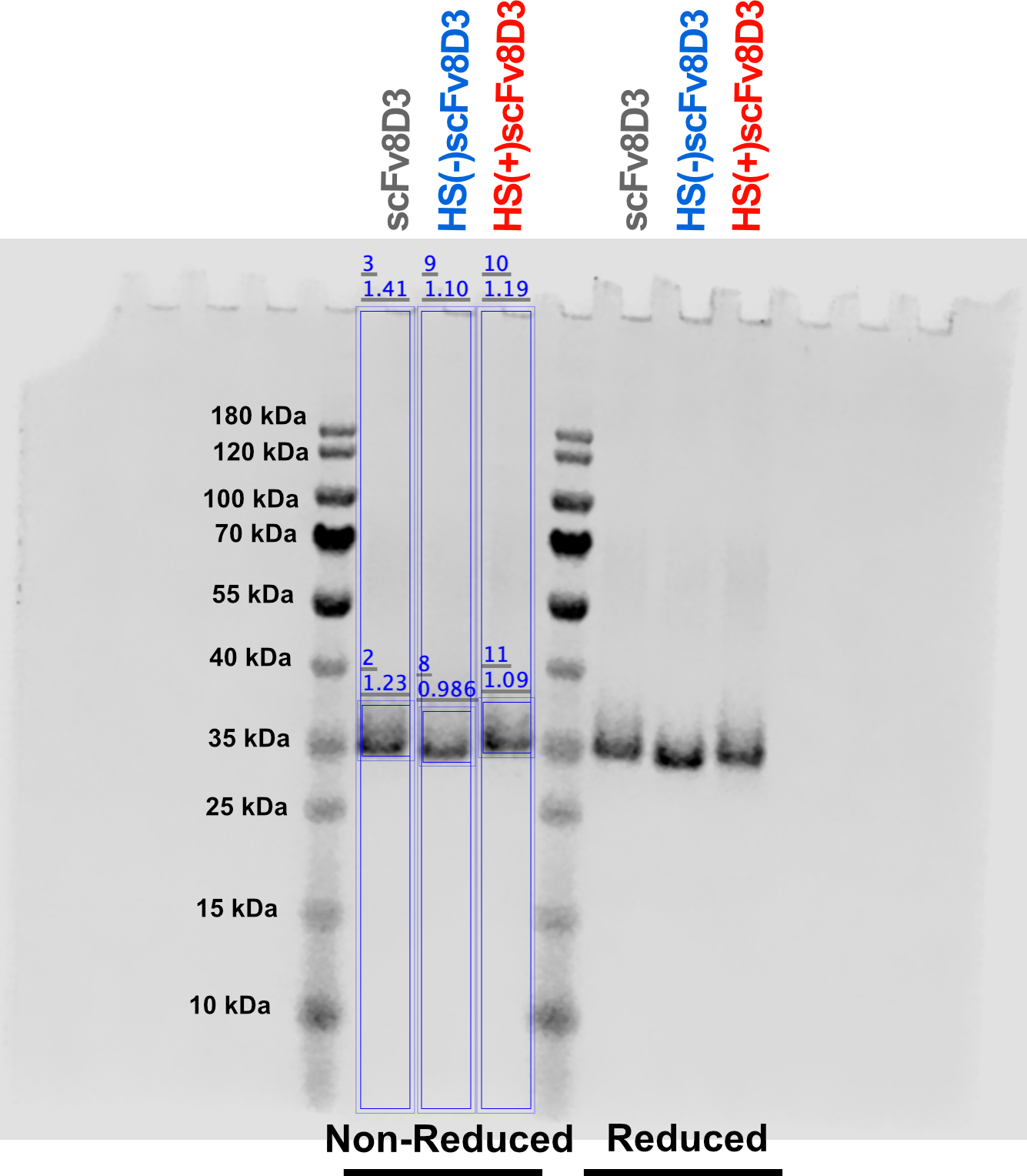


**Figure S1. scFv constructs purity analysis with Image Studio software.** SDS-PAGE gel analysis of the purified scFv constructs in non-reducing (left) and reducing conditions (right). The purity of the scFvs was estimated to be approximately 87% for scFv8D3, 90% for HS(-)scFv8D3 and 92% for HS(+)scFv8D3 with Image Studio software (version 5.2.5). Estimation was done by measuring the intensities of scFv8D3, HS(+)scFv8D3 and HS(-)scFv8D3 bands (rectangle 2, 8 and 11 respectively) divided by the intensities of their corresponding lanes (rectangle 3, 9 and 10 respectively).


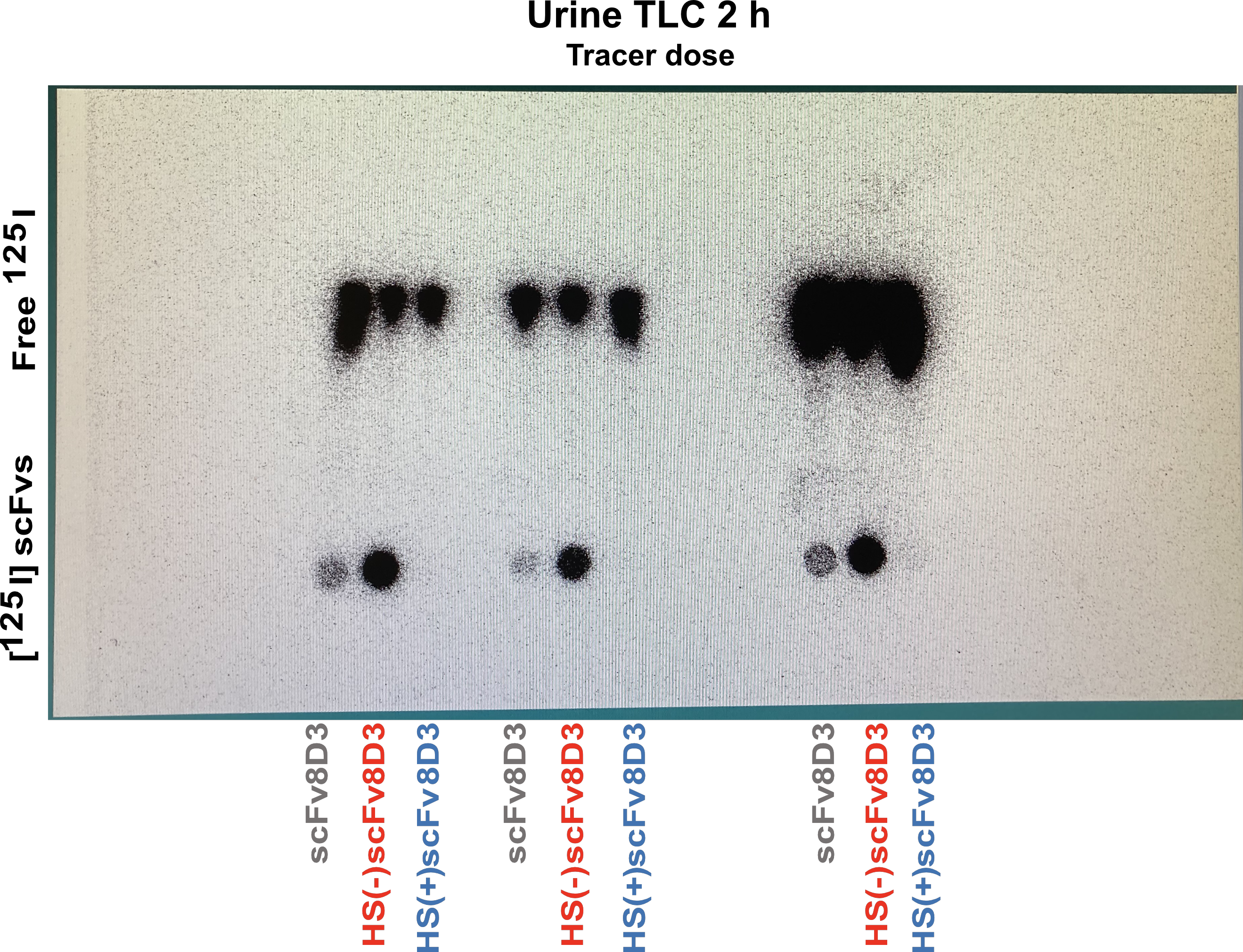


**Figure S2. Urine Thin-Layer Chromatography quality controls of [^125^I]scFvs constructs uncropped.** Uncropped membrane with 5 minutes exposure. The urine TLC quality control of the radiolabeled scFvs [^125^I]scFv8D3, (n= 3), [^125^I]HS(-)scFv8D3 (n=3) and [^125^I]HS(+)scFv8D3 (n=3) 2-hours post-injection.

**
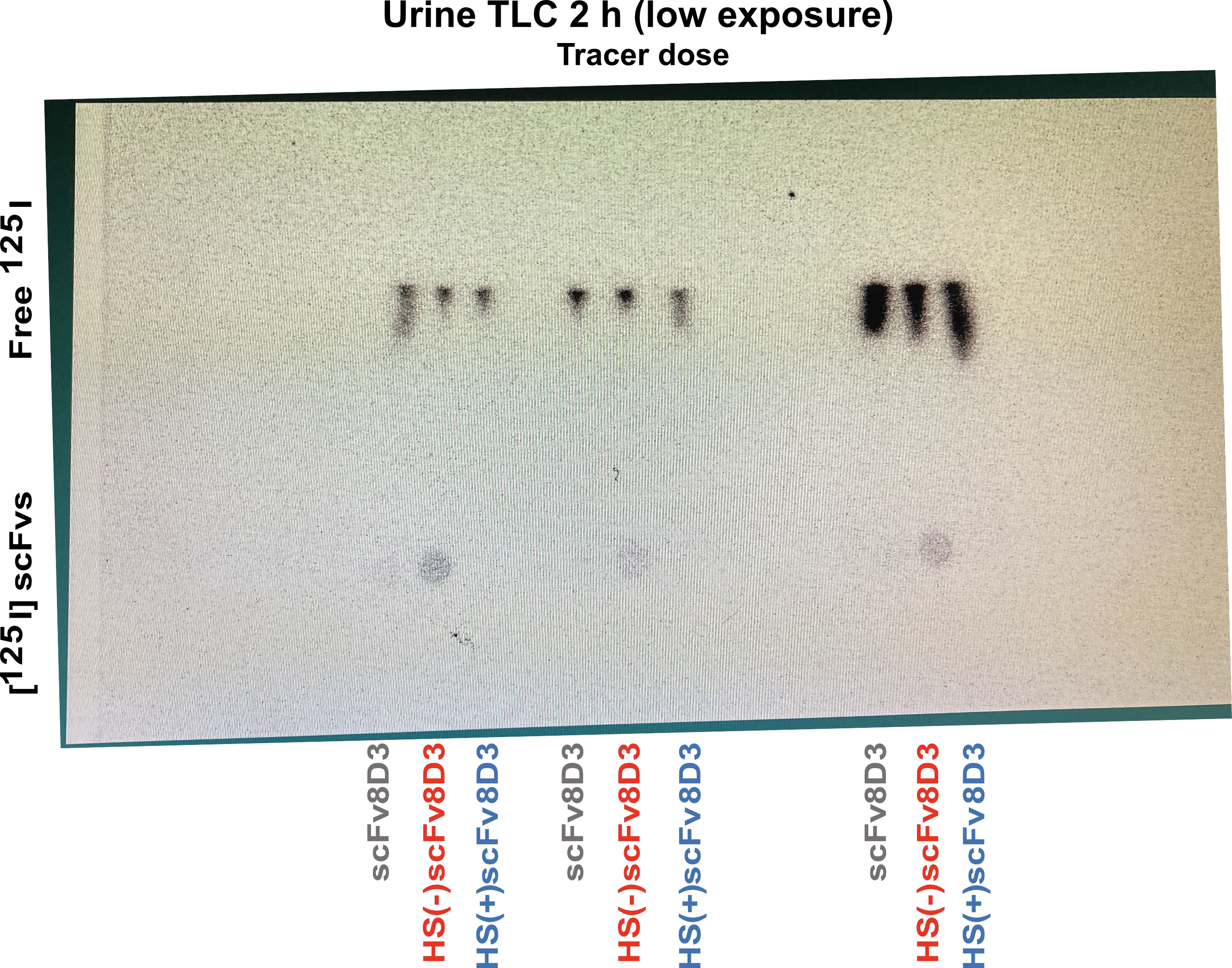
**

**Figure S3. Urine Thin-Layer Chromatography quality controls of [^125^I]scFvs constructs uncropped with low exposure.** Uncropped membrane with 1 minute exposure. The urine TLC quality control of the radiolabeled scFvs [^125^I]scFv8D3, (n= 3), [^125^I]HS(-)scFv8D3 (n=3) and [^125^I]HS(+)scFv8D3 (n=3) 2-hours post-injection.

Blood and peripheral biodistribution of scFv8D3 and HS mutants

In each *in vivo* experiment we analyzed the peripheral biodistribution of the three [I^125^]scFvs. However, since the *in vivo* stability of radiolabeled scFvs was diminished, the signal measured in the periphery derives only partly from the radiolabeled protein constructs (starting at roughly 50% at 2-3 hours post injection and decreasing with time, Figure 5), and the rest of the signal comes from free iodine-125 and partly degraded constructs.

Blood biodistribution of tracer dose of scFv8D3 and HS mutants

The distribution in blood, plasma and blood pellet of tracer doses (0.3 nmol/kg) of the three radiolabeled scFvs was similar at both the 2 and the 48-hour time-points (Figure S4).


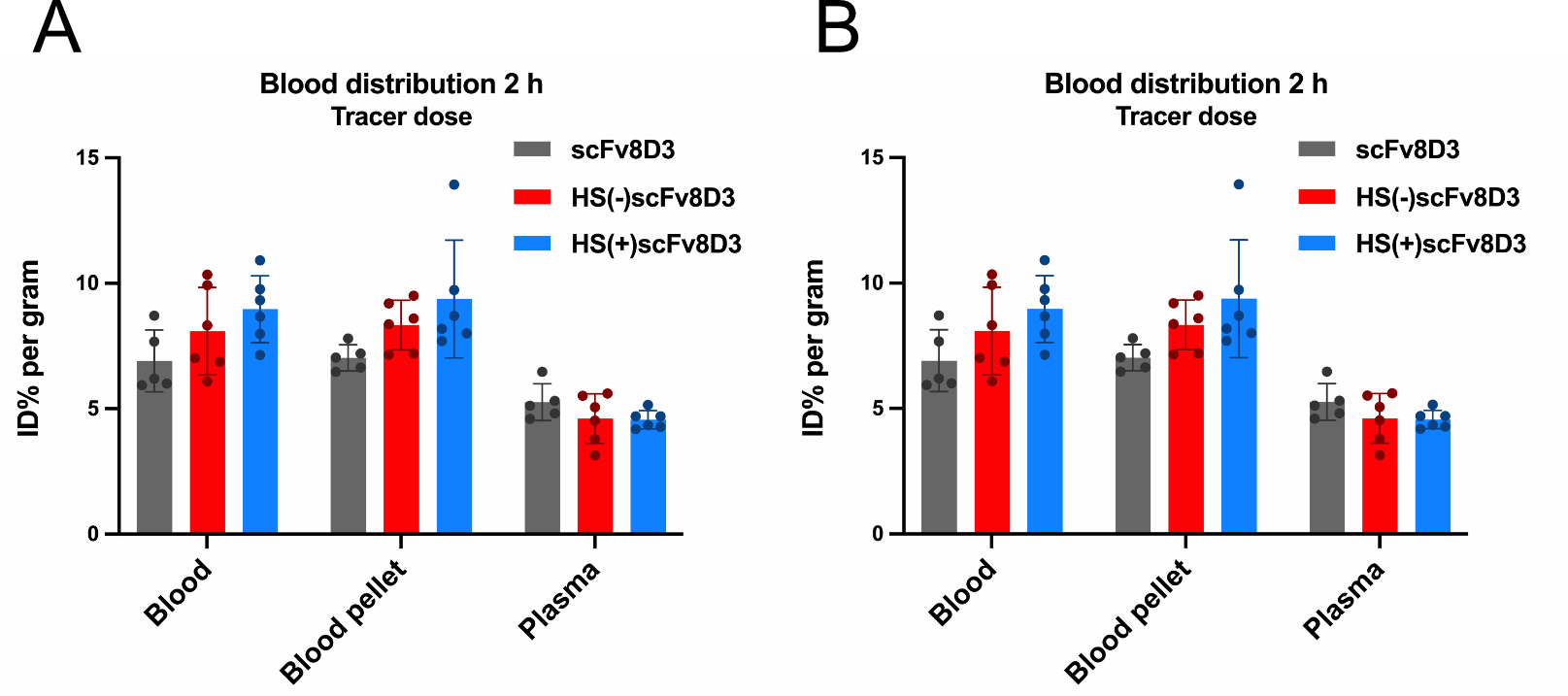


**Figure S4. Blood distribution of tracer doses of [^125^I]scFvs.**

The blood distribution of a tracer doses (0.3 nmol/kg) of the three scFv constructs was investigated by radiolabeling and measuring the activity 2 hours and 48 hours post-injection, presented as of %ID per gram sample. The distribution in blood, blood pellet and plasma were similar for all three [^125^I]scFvs. (A). 2-Hour *ex-vivo* blood distribution. [^125^I]scFv8D3 (n=5), [^125^I]HS(-)scFv8D3 (n=6) and [^125^I]HS(+)scFv8D3 (n=6). (B). 48-hour *ex vivo* blood distribution. [^125^I]scFv8D3 (n=8), [^125^I]HS(-)scFv8D3 (n=8) and [^125^I]HS(+)scFv8D3 (n=8). Results are presented as mean ±SD.

Blood distribution of therapeutic dose of scFv8D3 and HS mutants

The distribution in blood, plasma and blood pellet of therapeutic doses (30 nmol/kg) of the three radiolabeled scFvs was similar at the 24-hour time-point (Figure S5).


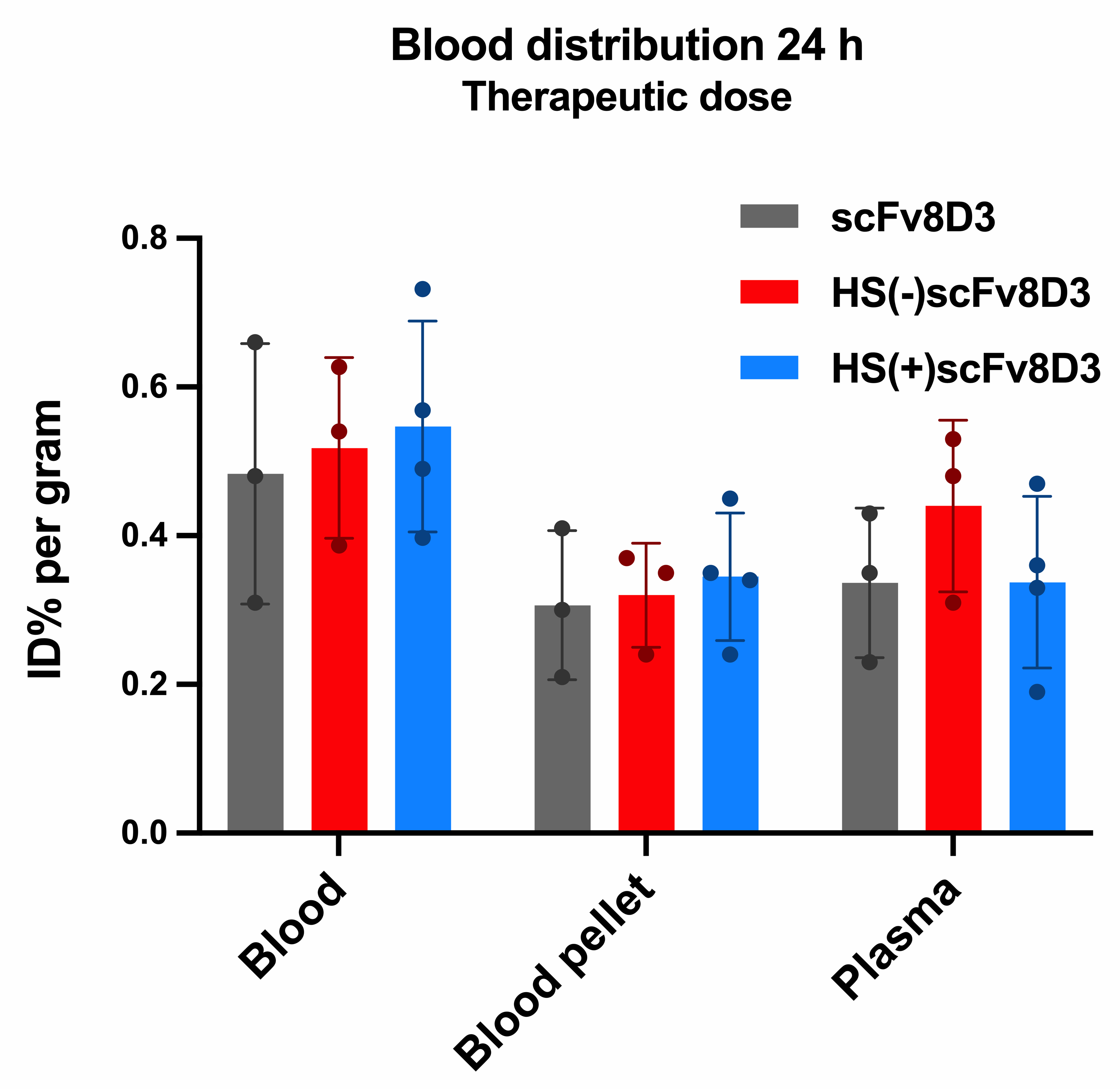


**Figure S5. Blood distribution of therapeutic doses of [^125^I]scFvs.** The blood distribution of therapeutic doses (30 nmol/kg) of the three scFv constructs was investigated by radiolabeling and measuring the activity 24 hours post-injection, presented as of %ID per gram sample. The blood distribution of a therapeutic dose of all three [^125^I]scFvs was similar 24 hours post injection. [^125^I]scFv8D3 (n=3), [^125^I]HS(-)scFv8D3 (n=3) and [^125^I]HS(+)scFv8D3 (n=4). Results are presented as mean ±SD.

Peripheral biodistribution of tracer dose of scFv8D3 and HS mutants

The peripheral distribution of tracer doses (0.3 nmol/kg) of the three radiolabeled scFvs was similar at both the 2 hour (Figure S6A) and the 48-hour time-points (Figure S6B). The peripheral distribution was highest in the thyroid, spleen and kidney and decreasing between the time points (Figure S6).


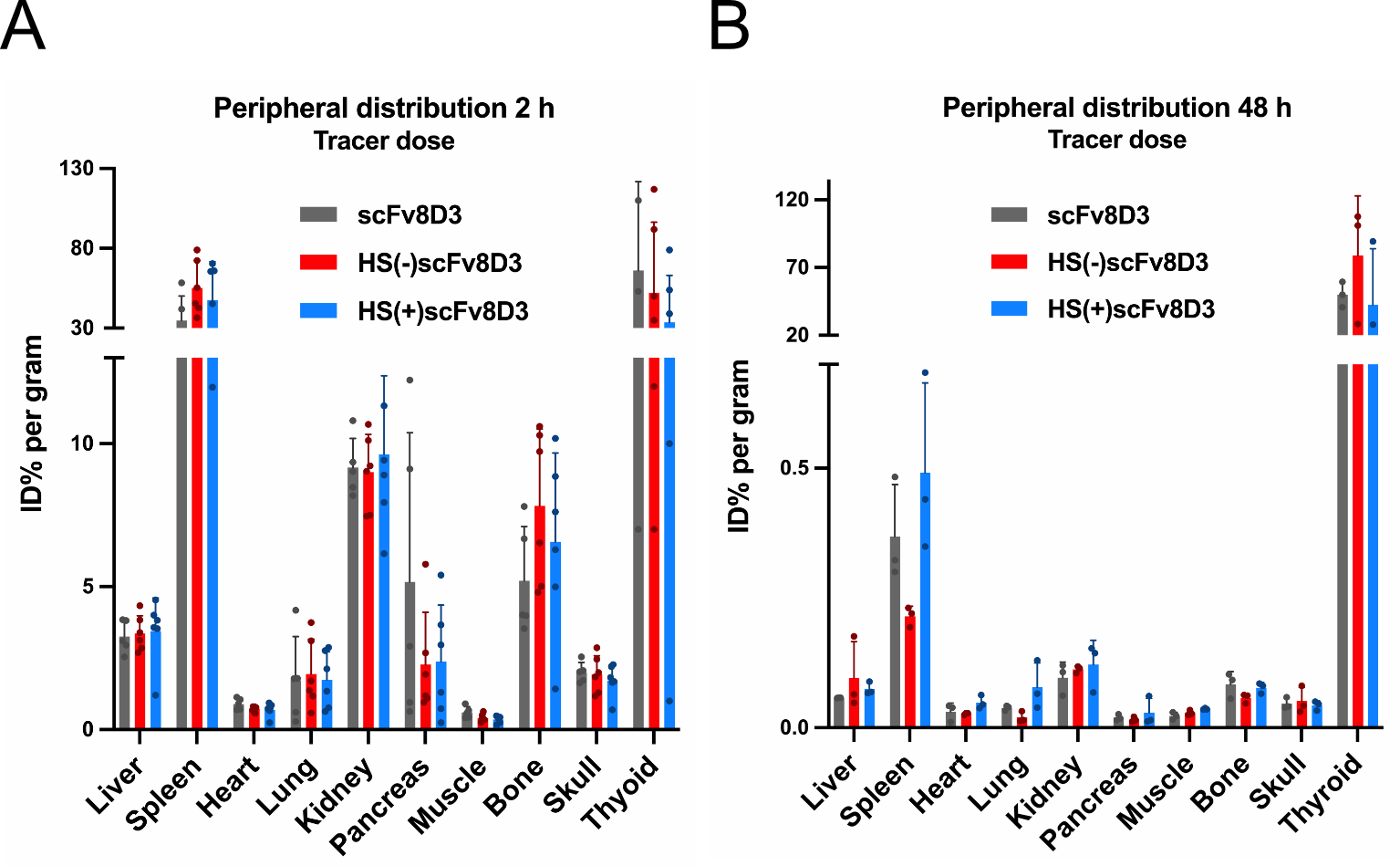


**Figure S6. Peripheral distribution of tracer doses of [^125^I]scFvs.** The peripheral distribution of tracer doses (0.3 nmol/kg) of the three scFv constructs was investigated by radiolabeling and measuring the activity 2 hours and 48 hours post injection, presented as of %ID per gram organ. (A). The peripheral distribution of a tracer dose of all three [^125^I]scFvs. The results are pooled from two separate ex-vivo experiments. [^125^I]scFv8D3 (n=5), [^125^I]HS(-)scFv8D3 (n=6) and [^125^I]HS(+)scFv8D3 (n=6) (B). The peripheral distribution of a tracer dose of all three [^125^I]scFvs. [^125^I]scFv8D3 (n=3), [^125^I]HS(-)scFv8D3 (n=3) and [^125^I]HS(+)scFv8D3 (n=3). Results are presented as mean ±SD.

Peripheral biodistribution of therapeutic dose of scFv8D3 and HS mutants

The peripheral of therapeutic doses (30 nmol/kg) of the three radiolabeled scFvs was similar at the 24-hour time-point (Figure S7). The peripheral distribution was highest in the thyroid, spleen and kidney (Figure S7).


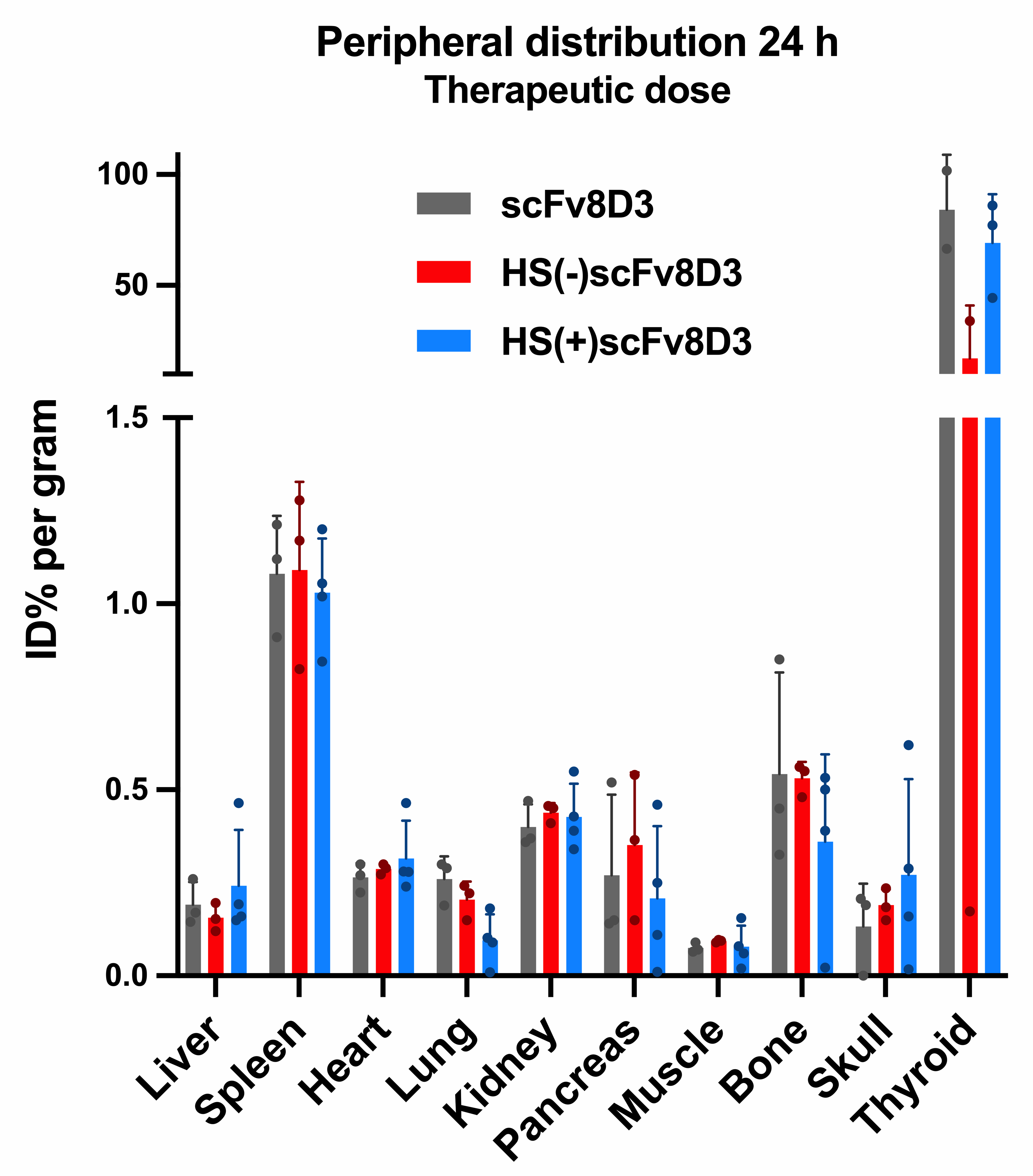


**Figure S7. Peripheral distribution of therapeutic doses of [^125^I]scFvs.** The peripheral distribution of therapeutic doses (30 nmol/kg) of the three scFv constructs was investigated by radiolabeling and measuring the activity 24 hours post-injection, presented as of %ID per gram organ. The blood distribution of a therapeutic dose of all three [^125^I]scFvs. [^125^I]scFv8D3 (n=3), [^125^I]HS(-)scFv8D3 (n=3) and [^125^I]HS(+)scFv8D3 (n=4). Results are presented as mean ±SD.

Estimating the contribution of free iodine-125 to radioactive signal of [^125^I]scFvs

To estimate the contribution of free iodine-125 (^125^I) to the radioactive signal measured in the in the *ex vivo* [^125^I]scFv brain uptake experiments calculations were performed. To do the calculations the brain penetrance of ^125^I was assumed to be non-saturable as indicated by its relatively constant brain-to-plasma ratio (ranging from 0.01 to 0.10) and brain-to-blood ratio (ranging from 0.01 to 0.07). Further, the brain penetrance of ^125^I was assumed to be correlated with the plasma concentration. To approximate the free ^125^I plasma concentration during the scFv *ex vivo* brain uptake experiments (Figure 6 and 7) the blood distribution data (Figure S2 and S3) from the scFv brain uptake experiments was used together with data from the *in vivo* plasma stability experiment (Figure 5) was used. Based on this, together with ^125^I brain penetrance data (Figure 11), the brain penetrance of the free iodine-125 plasma concentration during the *ex vivo* scFv brain uptake experiments was estimated (Table S1-, S2- and S3). At the 2h time point the contribution of free ^125^I was calculated to be 2.97%, 2.05% and 2.90% of the signal for [^125^I]scFv8D3, [^125^I]HS(-)scFv8D3 and [^125^I]HS(+)scFv8D3 respectively (Table S1). At the 24h time point it was calculated to be 2.54%, 4.00% and 3.38% of the signal for [^125^I]scFv8D3, [^125^I]HS(-)scFv8D3 and [^125^I]HS(+)scFv8D3 respectively (Table S2). At the 48h time point it was calculated to be ≤ 7.00%, 7.00% and 6.125% of the signal for [^125^I]scFv8D3, [^125^I]HS(-)scFv8D3 and [^125^I]HS(+)scFv8D3, respectively (Table S3).

**Table S1** Estimation of free ^125^I contribution to radioactive signal of [^125^I]scFvs at 2-hour time point

|  | Plasma concentration at 2-hours including free ^125^I for the [^125^I]scFvs.  (%ID/g plasma) | Ratio of [^125^I]scFv to  free ^125^I at 3-hours | Calculated free  ^125^I plasma concentration in [^125^I]scFv brain uptake experiment  ⇒ Calculated by multiplying the ratio of free ^125^I with the [^125^I]scFv %ID/g plasma concentration in the brain uptake experiments  (%ID/g plasma) | Brain concentration at 2-hours in brain uptake experiments  (%ID/g brain tissue) | Calculated percentage of ^125^I signal contribution uptake of [^125^I]scFv in brain uptake experiment  ⇒ Calculated by dividing the free ^125^I %ID/g plasma concentration in brain uptake experiments with the ^125^I %ID/g plasma concentration and then multiplying with the ^125^I %ID/g brain tissue yielding a calculated free ^125^I %ID/g brain concentration in the [^125^I]scFv brain uptake experiments, which is then finally dividing that by the measured [^125^I]scFv %ID/g brain tissue in the brain uptake experiments.  (%) |
| --- | --- | --- | --- | --- | --- |
| ^125^I | 4.42 | N/A | N/A | 0.05 | N/A |
| [^125^I]scFv8D3 | 5.26 | 0.61 | 2.05 | 0.78 | 2.97 |
| [^125^I]HS(-)scFv8D3 | 4.61 | 0.65 | 1.61 | 0.89 | 2.05 |
| [^125^I]HS(+)scFv8D3 | 4.56 | 0.42 | 2.64 | 1.00 | 2.90 |

**Table S2** Estimation of free ^125^I contribution to radioactive signal of [^125^I]scFvs at 24-hour time point

|  | Plasma concentration at 24-hours including free ^125^I for the [^125^I]scFvs.  (%ID/g plasma) | Ratio of [^125^I]scFv to  free ^125^I at 24-hours | Calculated free  ^125^I plasma concentration in [^125^I]scFv brain uptake experiment  ⇒ Calculated by multiplying the ratio of free ^125^I with the [^125^I]scFv %ID/g plasma concentration in the brain uptake experiments  (%ID/g plasma) | Brain concentration at 24-hours in brain uptake experiments  (%ID/g brain tissue) | Calculated percentage of ^125^I radioactive signal contribution  ⇒ Calculated by dividing the free ^125^I %ID/g plasma concentration in brain uptake experiments with the ^125^I %ID/g plasma concentration and then multiplying with the ^125^I %ID/g brain tissue yielding a calculated free ^125^I %ID/g brain concentration in the [^125^I]scFv brain uptake experiments, which is then finally dividing that by the measured [^125^I]scFv %ID/g brain tissue in the brain uptake experiments.  (%) |
| --- | --- | --- | --- | --- | --- |
| ^125^I | 0.11 | N/A | N/A | 0.004 | N/A |
| [^125^I]scFv8D3 | 0.34 | 0.38 | 0.21 | 0.30 | 2.54 |
| [^125^I]HS(-)scFv8D3 | 0.44 | 0.25 | 0.33 | 0.30 | 4.00 |
| [^125^I]HS(+)scFv8D3 | 0.34 | 0.24 | 0.26 | 0.28 | 3.38 |

**Table S3.** Estimation of free ^125^I contribution to radioactive signal of [^125^I]scFvs at 48-hour time point

|  | Plasma concentration at 48-hours including free ^125^I for the [^125^I]scFvs.  (%ID/g plasma) | Ratio of [^125^I]scFv to  free ^125^I at 48-hours | Calculated free  ^125^I plasma concentration in [^125^I]scFv brain uptake experiment  ⇒ Calculated by multiplying the ratio of free ^125^I with the [^125^I]scFv %ID/g plasma concentration in the brain uptake experiments  (%ID/g plasma) | Brain concentration at 48-hours in brain uptake experiments  (%ID/g brain tissue) | Calculated percentage of ^125^I radioactive signal contribution  ⇒ Calculated by dividing the free ^125^I %ID/g plasma concentration in brain uptake experiments with the ^125^I %ID/g plasma concentration and then multiplying with the ^125^I %ID/g brain tissue yielding a calculated free ^125^I %ID/g brain concentration in the [^125^I]scFv brain uptake experiments, which is then finally dividing that by the measured [^125^I]scFv %ID/g brain tissue in the brain uptake experiments.  (%) |
| --- | --- | --- | --- | --- | --- |
| ^125^I | 0.10 | N/A | N/A | 0.007 | N/A |
| [^125^I]scFv8D3 | 0.09 | 0.32 | 0.06 | 0.06 | 7.00 |
| [^125^I]HS(-)scFv8D3 | 0.09 | 0.20 | 0.07 | 0.07 | 7.00 |
| [^125^I]HS(+)scFv8D3 | 0.09 | 0.20 | 0.07 | 0.08 | 6.125 |
